# Extremophilic hemoglobins: The structure of *Shewanella benthica* truncated hemoglobin N

**DOI:** 10.1016/j.jbc.2025.108223

**Published:** 2025-01-24

**Authors:** Jaime E. Martinez Grundman, Thomas D. Schultz, Jamie L. Schlessman, Eric A. Johnson, Richard E. Gillilan, Juliette T.J. Lecomte

**Affiliations:** 1 T.C. Jenkins Department of Biophysics, Johns Hopkins University, Baltimore, Maryland, USA; 2 Chemistry Department, U.S. Naval Academy, Annapolis, Maryland, USA; 3 Department of Biology, Johns Hopkins University, Baltimore, Maryland, USA; 4 Center for High Energy X-ray Sciences, CHEXS, Ithaca, New York, USA

**Keywords:** hemoglobin, heme, NMR, SAXS, high pressure, oxygen binding, piezophile, quaternary structure

## Abstract

Truncated hemoglobins (TrHbs) have an ancient origin and are widely distributed in microorganisms where they often serve roles other than dioxygen transport and storage. In extremophiles, these small heme proteins must have features that secure function under challenging conditions: at minimum, they must be folded, retain the heme group, allow substrates to access the heme cavity, and maintain their quaternary structure if present and essential. The genome of the obligate psychropiezophile *Shewanella benthica* strain KT99 harbors a gene for a TrHb belonging to a little-studied clade of globins (subgroup 2 of group N). In the present work, we characterized the structure of this protein (SbHbN) with electronic absorption spectroscopy and X-ray crystallography and inspected its structural integrity under hydrostatic pressure with NMR spectroscopy and small-angle X-ray scattering. We found that SbHbN self-associates weakly in solution and contains an extensive network of hydrophobic tunnels connecting the active site to the surface. Amino acid replacements at the dimeric interface formed by helices G and H in the crystal confirmed this region to be the site of intermolecular interactions. High hydrostatic pressure dissociated the assemblies while the porous subunits resisted unfolding and heme loss. Preservation of structural integrity under pressure is also observed in nonpiezophilic TrHbs, which suggests that this ancient property is derived from functional requirements. Added to the inability of SbHbN to combine reversibly with dioxygen and a propensity to form heme *d*, the study broadens our perception of the TrHb lineage and the resistance of globins to extreme environmental conditions.

To date, a small number of microorganisms living at the low temperatures and high hydrostatic pressures of the deep marine subsurface have been collected and cataloged (1, 2, 3, 4). The features that distinguish these psychropiezophiles from organisms living at atmospheric pressure and moderate temperatures hold interest for basic research and biotechnological applications (5). Currently, the scant representation of psychropiezophiles in genetic databases and the technical challenges posed by their study limit progress toward a full appreciation of their specialized traits. While a larger and more diverse census of organisms from extreme environments would be ideal, comparison of currently known psychropiezophilic microbes to relatives from nonextreme or midextreme environs still offers original evolutionary insights along with clues on adaptation to cold and benthic biomes.

At the physiological level, psychropiezophiles must contend with several simultaneous sources of stress (6, 7). One defense against extremes of temperature and pressure is to stabilize cellular components with osmolytes, piezolytes, and chaperones (7, 8, 9). Redundant approaches are also necessary to address redox imbalance, a major hazard of life in cold and deep oceans. These approaches include the expression of multiple dedicated proteins, for example, catalases and superoxide dismutases (10), the synthesis of small antioxidant molecules (11), and in some instances, adjustments to the components of the respiratory system (12, 13, 14).

At the molecular level, analysis of the proteome of known psychropiezophilic microbes shows a bias in amino acid composition toward a subset of polar, small, and hydrophilic residues (15). This bias is expected to affect protein functional fitness: the size of internal cavities, the degree of hydration, and the flexibility of the structure (9, 16, 17). Few proteins from psychropiezophiles have been directly compared *in vitro* to their nonpiezophile counterparts, and these studies also highlight differences in quaternary structure and electrostatic surface, but overall paint a complicated, occasionally contradictory, picture of all factors (18, 19, 20, 21, 22, 23).

To expand the set of characterized proteins from psychropiezophiles, we turned to a heme protein from *Shewanella benthica* KT99, a deep-sea gammaproteobacterium originally found in the Tonga–Kermadec subduction zone. It is an obligate psychrohyperpiezophile thriving at 4 to 10 °C and pressures as high as 1000 atm (24, 25, 26). The genome of *S. benthica* KT99 has been sequenced (26). Among its predicted proteins are two hemoglobins (Hbs) of the truncated family (TrHb), referred to here as SbHbN and SbHbO. TrHbs originated from the ancestral Hb fold, which is thought to have appeared before the last universal common ancestor (27), and have become widespread in bacteria, archaea, unicellular eukaryotes, and plants (28). Multiple microbial members have been extensively studied through *in vitro* activity and structural determination (29). Thus, the TrHbs of *S. benthica* offer an opportunity to explore the function and structure of a primordial TrHb fold adapted to benthic conditions and to compare it against its well-studied nonpsychropiezophilic counterparts.

Although dioxygen storage is the anticipated function of many TrHbs, these proteins are *a priori* capable of a wide range of chemistries linked to the versatility of the heme group. As a consequence, the actual purpose of most TrHbs may vary from organism to organism and has remained generally elusive. However, some TrHbs readily process reactive nitrogen and oxygen species (29, 30, 31, 32), suggesting participation in the mitigation of these destructive molecules within the cell. Such a role is an attractive possibility for SbHbN and SbHbO.

In a previous study, we described the active site of SbHbN with bound heme *b* (iron–protoporphyrin IX, the metallocofactor of generic Hbs) and noted the facile modification of the protein under peroxide stress, identified as oxidation of heme *b* to heme *d* and covalent attachment of the heme or a heme derivative to the protein (33). The formation of heme *d* is intriguing for its involvement in complex enzymes such as clade 2 catalases (34, 35), which process H_2_O_2_ into O_2_ and H_2_O, and cytochrome *bd* oxidases (36), bacterial respiratory oxidases that carry out the reduction of O_2_ to H_2_O. In view of a potential role for SbHbN in oxidative stress management, we now compare it to the current cohort of amino acid sequences within its phylogenetic clade to extract signatures of psychropiezophily. We elaborate on the structural description of the protein as the first example of a large subgroup of TrHbs and investigate a single-site variant with modified peroxide sensitivity. We provide evidence that SbHbN self-associates in solution and confirm the location of the interface by site-directed mutagenesis. Finally, we subject the protein and variants to high hydrostatic pressure to mimic the behavior in the deep sea. In total, these investigations expand our view of the TrHb lineage with experimental data on a little-studied subgroup of Hbs and add to the discussion of the determinants of piezophily.

## Results and discussion

### TrHbN and SbHbN primary structures

**Figure 1:**
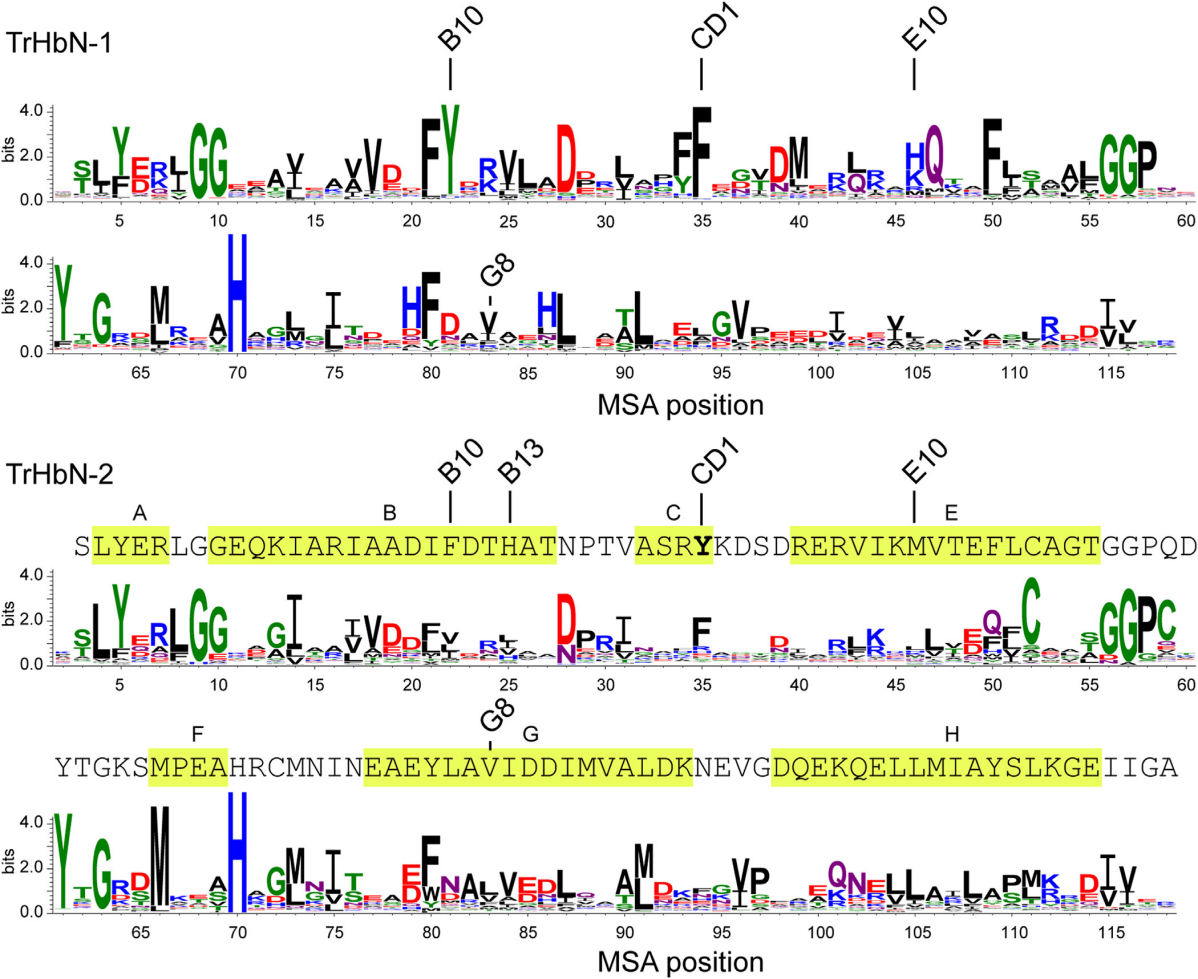
**Sequence logo plots** (40) **of the two subgroups of TrHbNs.** The reference numbering for the multiple sequence alignment (MSA) is from *Synechococcus* sp. PCC 7002 GlbN, a TrHbN-1 protein. The sequence of SbHbN is included above the TrHbN-2 logo plot and is one residue shorter than the consensus. Topological positions B10, B13, CD1, E10, and G8 are indicated. Helical regions detected in cyanomet S2SbHbN (Protein Data Bank ID: 8UGZ) are highlighted in *yellow*. Details of the procedure to generate the MSA are provided in the supporting information. S2SbHbN, *Shewanella benthica* strain KT99 TrHbN-2 (UniProt ID: A9DF82) with Cys51Ser and Cys71Ser replacements; TrHbN, truncated hemoglobin group N, also referred to as group 1 in the literature; TrHbN-1, truncated hemoglobin group N subgroup 1; TrHbN-2, truncated hemoglobin group N subgroup 2.

Phylogenetic analyses divide the TrHb family into four groups, labeled N, O, P, and Q (37). The N group is further split into two main subgroups (38), called here TrHbN-1 and TrHbN-2. To place SbHbN in context, we revisited our TrHbN alignment from 2006 with the increased number of sequences listed in the National Center for Biotechnology Information Conserved Domain Database (39), created a phylogenetic tree, and generated sequence logos (40) for each N subgroup (Figs. 1 and S1).

Key positions in the sequences of TrHbs have been identified through decades of TrHbO and TrHbN-1 studies (27). The conserved “proximal” histidine, located at topological position F8 following the myoglobin nomenclature (41), anchors the heme group in its pocket with a coordination bond to the iron. The residue at position E10, on the “distal” side of the heme, may coordinate the heme iron (42, 43, 44) and yield an endogenous hexacoordinate (6c) complex facilitating electron transfer steps. Residues at B10, CD1, E7, E11, and G8, which line the heme pocket, have a strong influence on the affinity for exogenous ligands (*e.g.*, O_2_) (27, 37, 45). Experimental and computational data lead to the conclusion that proteins with Tyr(B10), Gln(E7), and Gln(E11), which form a hydrogen bond network with exogenous ligand, tend to have high O_2_ affinity suitable for enzymatic activity, whereas those with hydrophobic residues at the same locations have low O_2_ affinity suitable for O_2_ transport and storage.

The sequence of SbHbN (Fig. 1), with hydrophobic residues at B10, E7, E11, and G8, not only conforms largely to the TrHbN-2 consensus but also shows notable deviations. A tyrosine occupies position CD1 where a phenylalanine is preferred. In SbHbN, we have found that Tyr(CD1)34 is implicated in the reactivity of the protein toward peroxides (33), likely through direct interaction with H_2_O_2_. At position B13, the consensus favors a hydrophobic residue; in contrast, SbHbN uses His(B13)24, which provides additional polarity, proton transfer potential, and hydrogen bonding capability to the distal side of the heme cavity. Thus, although SbHbN is a confirmed TrHbN-2, and TrHbN-2s, as a set, are expected to have low O_2_ affinity (37), the properties of this protein escape easy prediction. Interestingly, the second Hb of *S. benthica* KT99 (SbHbO, UniProt ID: A9D041) falls in the catalase–peroxidase functional type (37). SbHbN and SbHbO may play complementary roles in *S. benthica*.

Of practical significance are the two cysteines of SbHbN, Cys51(E16) and Cys71(F10). These residues are outside the heme cavity and too distant for the formation of an intramolecular disulfide bond. We replaced each with a serine to generate a pseudo-wildtype protein adequate for purification and study (33). All experiments were performed in this “S2SbHbN” background. Because of the presence in the heme pocket of Tyr(CD1)34 and Met(E10)45, the latter being a potential distal ligand to the iron, our first step in the characterization of the protein was to identify the coordination of the heme in ferric (Fe^3+^) and ferrous (Fe^2+^) SbHbN and inspect its ability to form a stable complex with O_2_.

### Heme coordination and ligand binding in S2SbHbN and the Y34F variant

**Figure 2:**
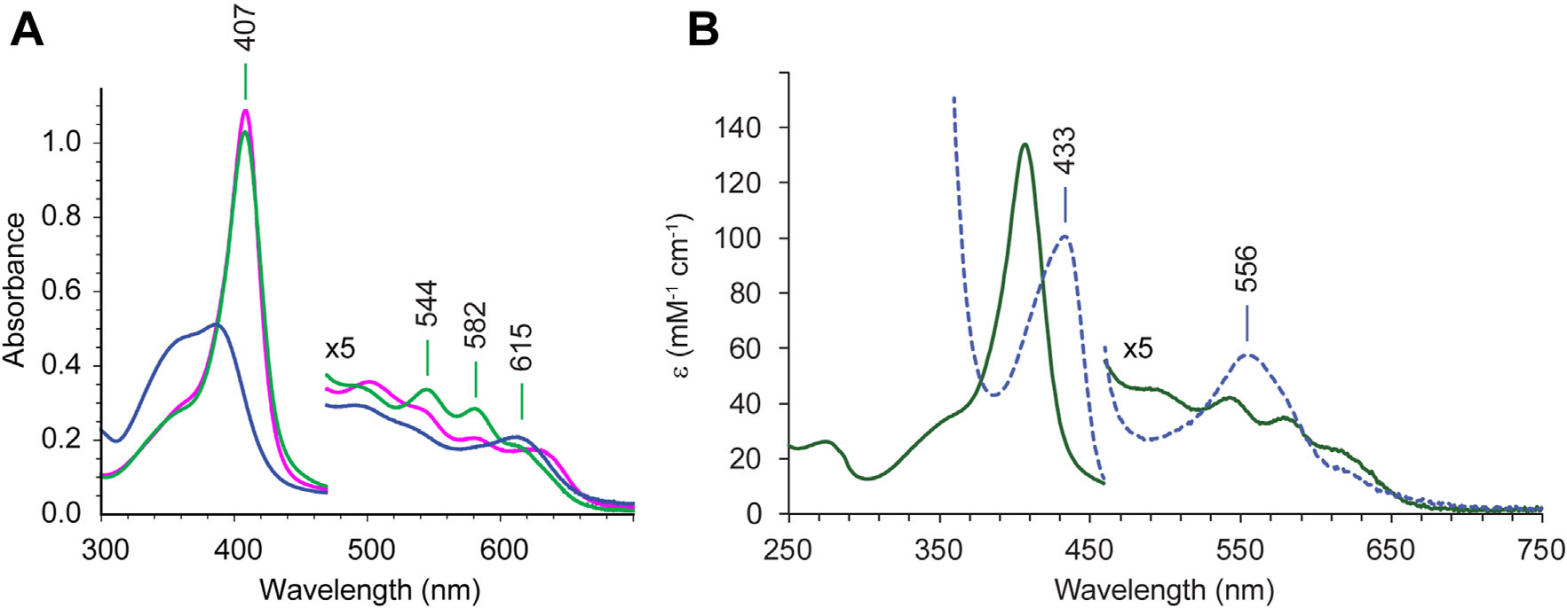
**Electronic absorption spectra of S2SbHbN.***A*, the ferric protein at pH 5.1 (*magenta*), 7.1 (*green*), and 11.4 (*blue*). At low pH, the appearance of the spectrum suggests a contribution from water coordination, whereas the spectrum between pH 7 and 10 is consistent with a hydroxide ligand. Above pH 10, the heme dissociates from the protein; below pH 5, the protein precipitates. *B*, the ferric (*green*) and ferrous (*blue, dashed*) states at neutral pH. S2SbHbN, *Shewanella benthica* strain KT99 TrHbN-2 (UniProt ID: A9DF82) with Cys51Ser and Cys71Ser replacements.

The electronic absorption spectrum of ferric S2SbHbN is shown in Figures 2*A*, S2 and S3. Spectral characteristics are listed in Tables S1 and S2. At neutral pH, the Soret band has a maximum at 407 nm. The spectrum in the visible region has maxima at 544 nm, 582 nm, and a shoulder at 615 nm. The shape is reminiscent of a spin 3/2 species or an equilibrium mixture of spin 1/2 and spin 5/2 species (46), presumably because of the coordination of the proximal histidine and a distal hydroxide ion (hydroxymet state). The ^1^H NMR spectrum (Fig. S4) exhibits broad lines between 25 and 60 ppm in support of this interpretation (47, 48). At pH below 6, protonation of the bound hydroxide converts the protein to the water-bound (aquomet) state. The pH response of the absorption spectrum is shown in Fig. S4.

**Figure 3:**
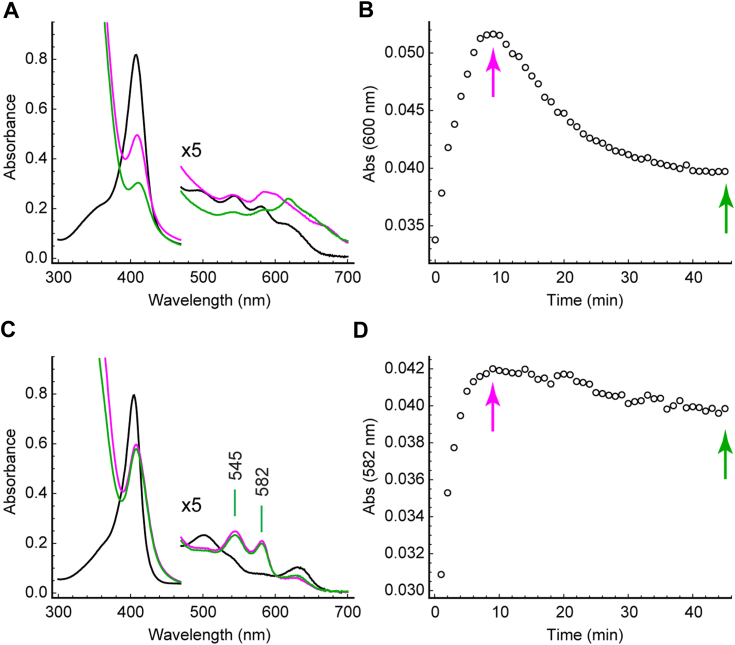
**Globin reduction and interaction with dioxygen.** (*A* and *B*) S2SbHbN; (*C* and *D*) Y34F S2SbHbN. The ferric proteins with no added ligand were mixed with the enzymatic Fd–FNR system (*black*). Addition of NADPH to initiate reduction resulted in spectral changes illustrated after 10 min (*magenta*) and 45 min (*green*). The *colored arrows* in (*B*) and (*D*) indicate the time stamps of the spectra in (*A*) and (*C*). S2SbHbN, *Shewanella benthica* strain KT99 TrHbN-2 (UniProt ID: A9DF82) with Cys51Ser and Cys71Ser replacements.

Reduction of S2SbHbN with sodium dithionite caused a red shift of the Soret band from 407 nm to 433 nm (Fig. 2*B*). The resulting spectrum resembles that of *Mycobacterium tuberculosis* HbN (a TrHbN-1) (49) and other 5c deoxy TrHbNs rather than that of the K(E10)M variant of *Chlamydomonas reinhardtii* THB1 (a TrHbN-1) in which methionine coordination was observed in the ferrous and not the ferric state (50). Reduction of the ferric protein was also performed with an enzymatic system to produce the oxy complex (51). Figure 3, *A* and *B,* shows the result of incubating S2SbHbN with the enzyme mixture prior to adding NADPH, then after 10 min of exposure to excess NADPH. In this manual mixing experiment, the typical globin oxy bands at ∼581 nm (α) and ∼545 nm (β) were not detected. The Soret band settled at 416 nm after several hours, and the end spectrum had broad features suggestive of heme damage perhaps through the formation of a reactive ferryl (Fe(IV)=O) species.

These results and the previously investigated influence of Tyr34 in the reactivity of S2SbHbN (33) convey singular importance to position CD1. To explore this position further, the same experiments were performed with the Tyr(CD1)34Phe variant. Figures 3*C* and S2 present the neutral pH spectrum of the ferric state, which corresponds to a water-bound complex. NMR data concur with a high-spin species typical of aquomet globins with hyperfine shifted resonances appearing downfield from 80 ppm (Fig. S4). This water-bound state dominates between pH 5 and 10.8 (Fig. S3), showing that Tyr(CD1)34 is more apt at stabilizing the anion (OH^−^) than the neutral ligand (H_2_O). With respect to oxygen binding (Fig. 3, *C* and *D*), the variant developed the expected α and β bands upon aerobic enzymatic reduction. These simple spectral experiments suggest that TrHbN-2s with Tyr(CD1)34 have dioxygen binding and reactivity properties different from those with Phe(CD1). The reason that the Tyr(CD1)34Phe replacement is consequential stems either from distortion of the heme pocket or the in-place absence of the hydroxyl group, the latter explanation simplifying any mechanistic interpretation of heme *d* formation. To resolve this issue, we proceeded with a structural comparison of S2SbHbN and its Y34F variant. We first discuss briefly distinct features of TrHbN-2s that we have not addressed in prior work (33) and inspect the properties of the Y34F variant. Additional interest in this variant is its closer relationship to the majority of TrHbN-2s (Fig. 1).

### The structure of cyanomet S2SbHbN

#### Topology of the globin domain

Most ferric globins bind cyanide tightly to the iron and form a stable cyanomet state amenable to structural determination. S2SbHbN is no exception (Fig. S5) despite the absence of a tyrosine at position B10. Cyanomet S2SbHbN crystallized in the P2_1_2_1_2_1_ space group (33) (Table S3 summarizes crystallographic data and refinement statistics). The asymmetric unit contains four chains. Of these, chain A exhibits well-defined electron density over the entire molecule, whereas the other three chains are less clearly traceable between Pro57 and Tyr60, at the start of the EF loop. Across TrHbNs, the EF loop is a flexible element prone to rearrangement and marked by elevated *B*-factors in X-ray diffraction models or missing resonances in NMR spectra (52). The definition of the Pro57–Tyr60 region in chain A is attributed to lattice contacts with helix A of a neighboring chain B.

**Figure 4:**
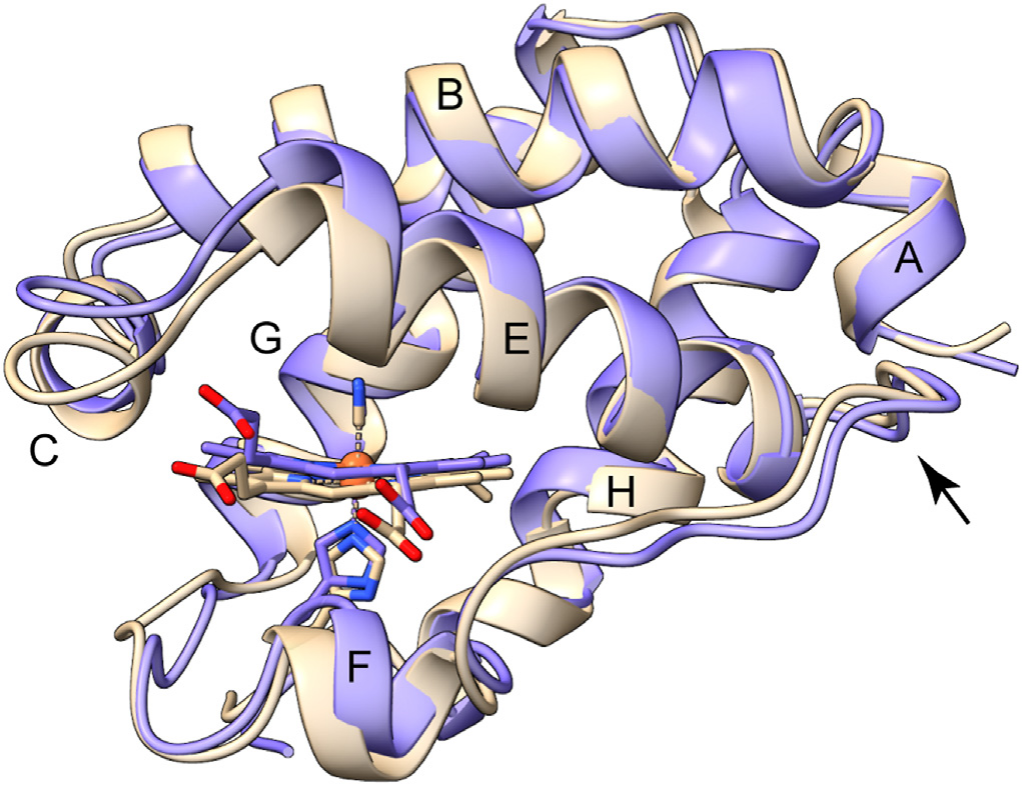
**Ribbon diagram of cyanomet S2SbHbN (Protein Data Bank ID:****8UGZ****chain A, *blue*) and *Mycobacterium tuberculosis* Y(B10)F/Q(E11)V HbN (Protein Data Bank ID:****2Gl3****chain A, *tan*** (94)**).** The *M. tuberculosis* structure has a 13-residue pre-A region not shown. The *arrow* points to residues 57 to 59 in the flexible EF loop. The superposition has an RMSD of 1.4 Å over 116 CA pairs. S2SbHbN, *Shewanella benthica* strain KT99 TrHbN-2 (UniProt ID: A9DF82) with Cys51Ser and Cys71Ser replacements.

The secondary and tertiary structures match closely those of relatives from subgroup 1 (Fig. 4). As is often observed in TrHbs, the A and F helices of S2SbHbN span only four residues. The H helix, which begins with residue 97, extends to residue 113, four residues short of the C terminus, and bends at residue Leu(H12)110 where bifurcated and side-chain/main-chain hydrogen bonds distort the geometry. As will be shown, this region impacts the assembly of the higher order structure. Throughout each chain, several intrahelical contacts contribute to the maintenance of the fold. A summary of helix capping interactions is provided in the supporting information.

**Figure 5:**
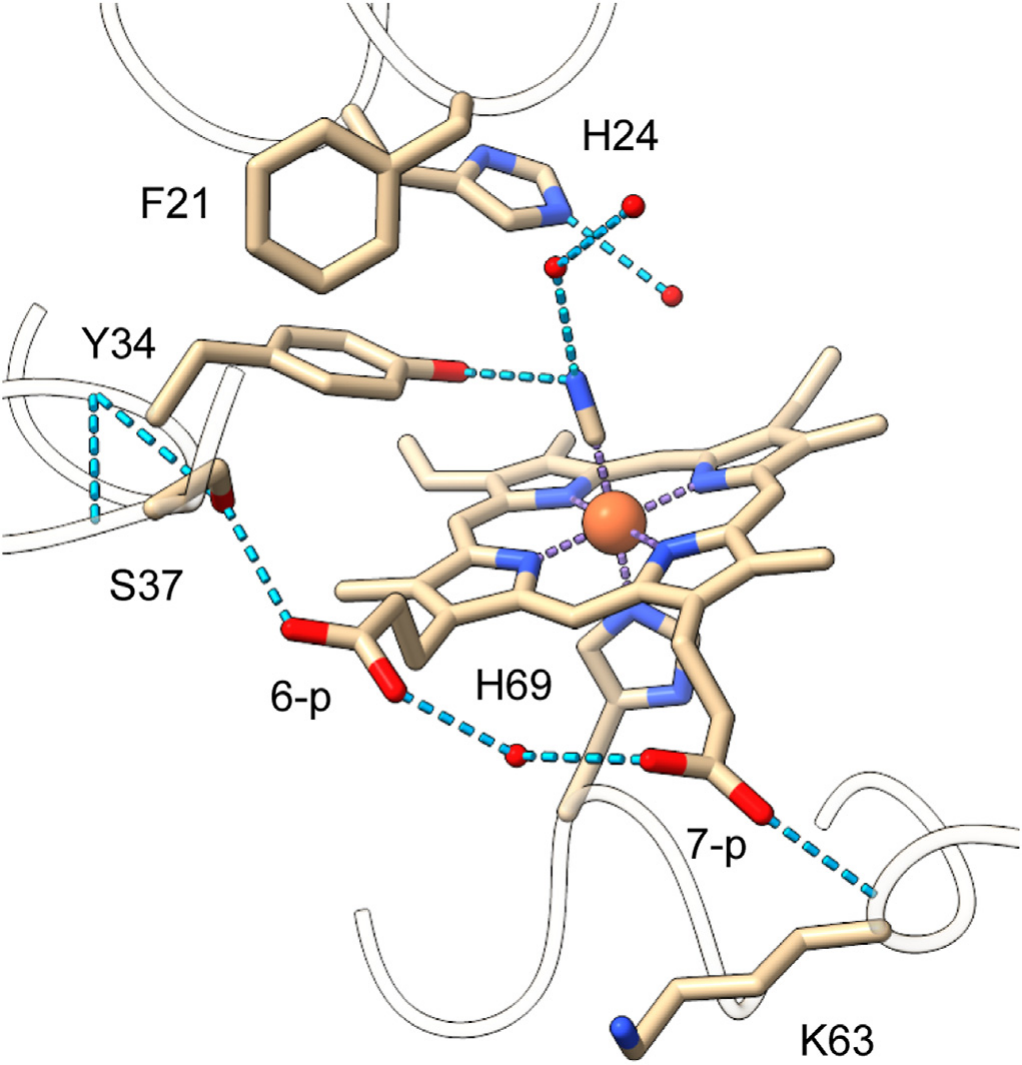
**The distal pocket in cyanomet S2SbHbN (Protein Data Bank ID:****8UGZ****, chain D).** Tyr(CD1)34 is within hydrogen-bonding distance of the bound cyanide ion. His(B13)24 is the only other polar residue in the pocket. Phe(B10)21 and His(F8)69 are shown. The heme 6- and 7-propionates are labeled 6-p and 7-p, respectively. S2SbHbN, *Shewanella benthica* strain KT99 TrHbN-2 (UniProt ID: A9DF82) with Cys51Ser and Cys71Ser replacements.

When comparing the heme pocket to that of a TrHbN-1 with close sequence resemblance (Y(B10)F/Q(E11)V *M. tuberculosis* HbN, Protein Data Bank [PDB] ID: 2GL3), we note excellent correspondence for the position of the heme group and side chains, except that Phe(B10) adopts the t rotameric state in structure 8UGZ and the g^−^ rotameric state in structure 2GL3. This allows an ordered water molecule to be positioned within hydrogen-bonding distance of bound cyanide. In 8UGZ, the hydroxyl group of Tyr(CD1)34 completes a three-member hydrogen-bonding network (Fig. 5). All four chains display similar distal pocket interactions.

**Figure 6:**
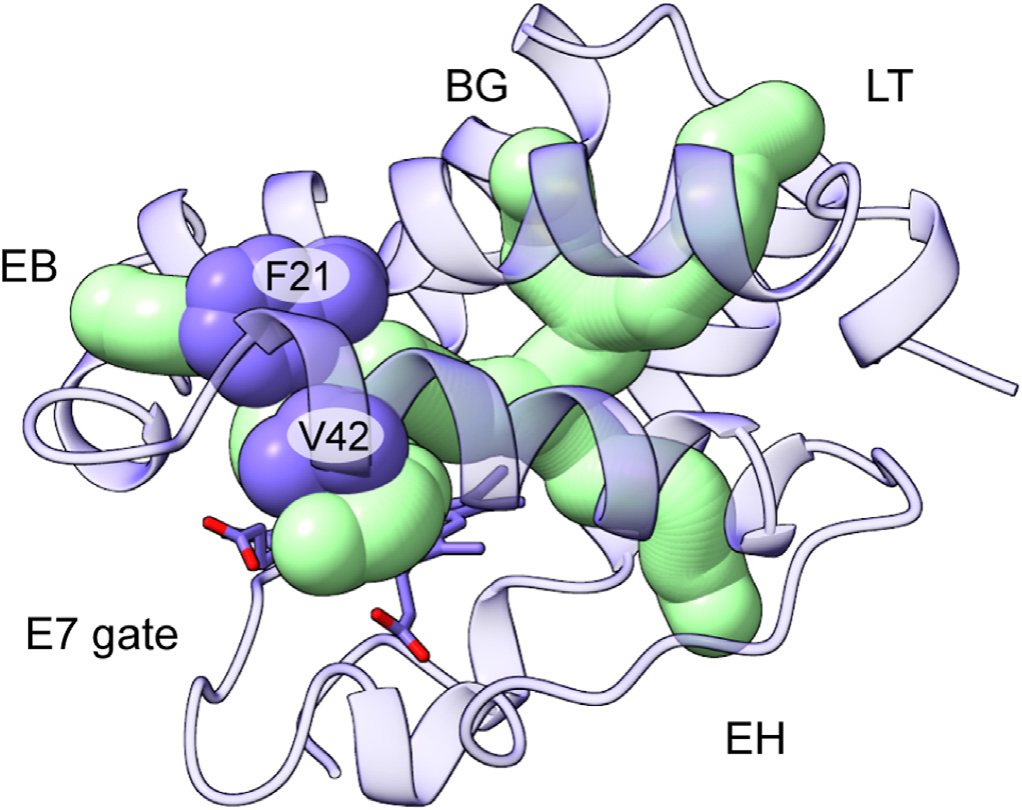
**Tunnels in the structure of cyanomet S2SbHbN (Protein Data Bank ID:****8UGZ****, chain A) shown as interconnected *green tubes* (1 Å bottleneck radius).** Labeled tunnels include those described for TrHbN-1s (37). LT, long tunnel; EH, E/H tunnel; EB, E/B tunnel; and E7 gate. Val(E7)42 and Phe(B10)21 are shown with *spheres* and the heme with *sticks*. S2SbHbN, *Shewanella benthica* strain KT99 TrHbN-2 (UniProt ID: A9DF82) with Cys51Ser and Cys71Ser replacements.

The chemistry of TrHbs is intimately dependent on the accessibility of the heme group by solvent and ligands (37, 53). In SbHbN, channels lined with hydrophobic residues and punctuated with intervening bottlenecks connect the distal pocket to the surface (Fig. 6). A long tunnel extends from the GH turn to the heme and has four short branches exiting between helices B and G, E and B, E and H, and E and the heme. Interestingly, the t rotameric state of Phe(B10)21 leaves the EB branch open. Except for the ordered water molecules shown in Figure 5 and occasional molecules at the extremities of the branches, these tunnels contain no crystallographic water molecules, suggesting that backbone and side-chain fluctuations facilitate rapid transit of small molecules through the protein.

Further analysis was performed with CAVER (54) to locate packing defects within the static monomers. All four chains revealed the same six cavities (Fig. S6). The total average void volume is ∼120 Å^3^, a value within the range spanned by similarly sized proteins (55). Most mesophilic TrHbN-1s have void volumes lower than what is seen for SbHbN; for example, *Chlamydomonas eugametos* GlbN (PDB ID: 1DLY) has three cavities for a total volume of ∼50 Å^3^. Thus, although packing defects are thought to increase the sensitivity of proteins to pressure, SbHbN does not appear better packed than its relatives.

#### Assembly in the crystal

**Figure 7:**
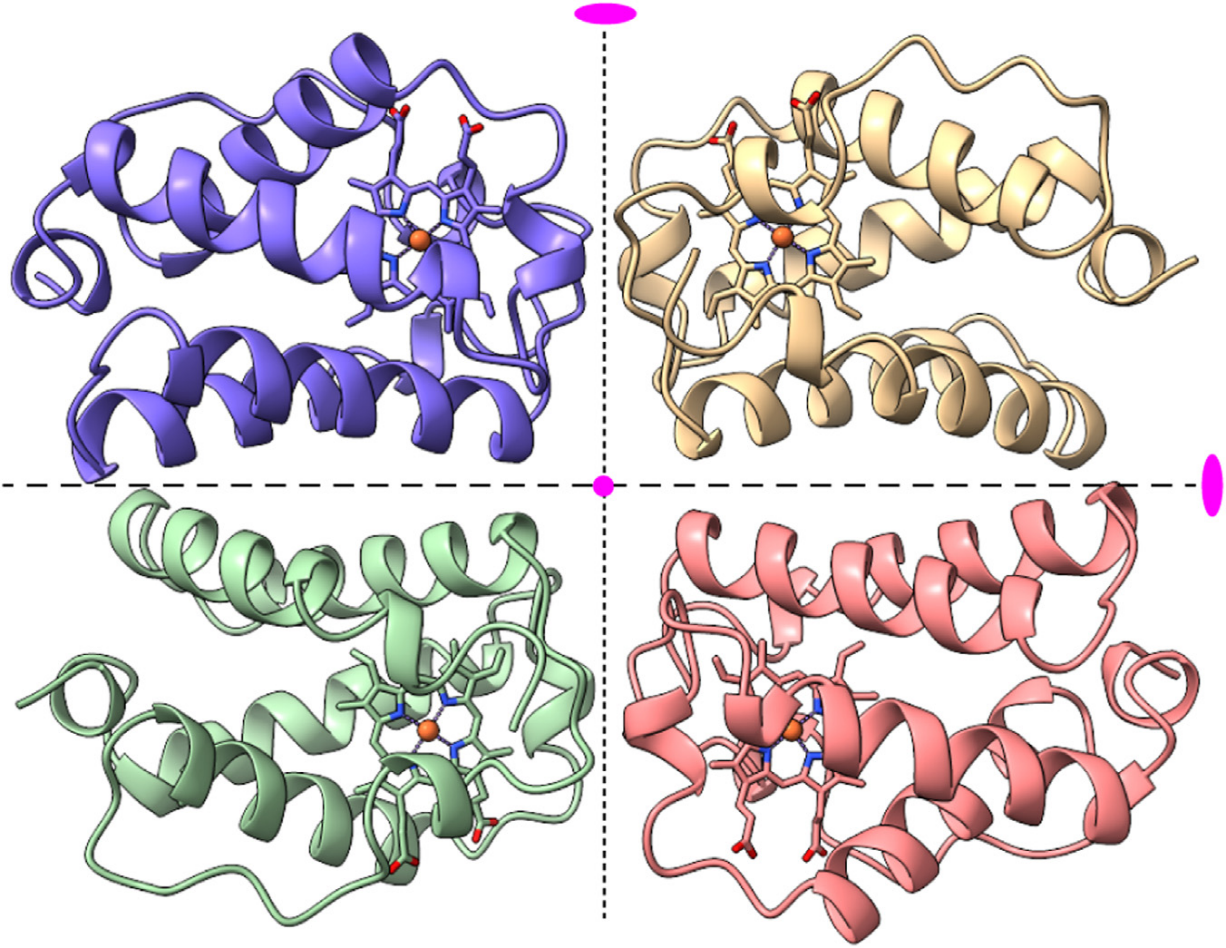
**The asymmetric unit of cyanomet S2SbHbN (PDB ID****8UGZ****).** The four chains match each other to an average RMSD of 0.3 Å (116 CA atom pairs), the deviation largely caused by variability in the EF loop. The *dashed and dotted lines* indicate two of the three *C*_2_ axes of the dihedral assembly. The third is perpendicular to the page (*magenta dot* in the center). The heme groups are represented with *sticks*. S2SbHbN, *Shewanella benthica* strain KT99 TrHbN-2 (UniProt ID: A9DF82) with Cys51Ser and Cys71Ser replacements.

The four chains composing the asymmetric unit are organized with dihedral symmetry as shown in Figure 7. The tetrameric arrangement has two interfaces, one involving the G and H helices of two chains (above and below the *dashed line*) and the other involving the C and FG loop of two chains (left and right of the *dotted line*). Interheme distances across the G–H and C–FG interfaces are 30 Å and 22 Å, respectively. The center of the tetramer is occupied by several water molecules and Asn(G1)75 in the FG loop of each of the chains. Steric clashes between chains A(C) and D(B) force the Asn(G1)75 side chains to adopt two alternate locations in the tetramer (Fig. S7). PISA analysis (56) identifies the G–H interface, which buries a mere 720 Å^2^, as stable and hydrophobic. This suggests that the protein may populate a G–H dimer at solution concentrations used for our experiments. The C–FG interface (560 Å^2^) is attributed to weak crystal contacts. To our knowledge, the G–H dimer of S2SbHbN represents a novel Hb subunit arrangement (57).

### The structure of Y34F S2SbHbN

Depending on buffer conditions, crystals of cyanomet Y34F S2SbHbN either lost cyanide over the course of several days or retained it through cryo-temperature data collection. Cyanomet Y34F S2SbHbN crystallized in the same space group as cyanomet S2SbHbN and remained in that space group after cyanide loss. The cyanide-free structure was refined to 2.0 Å (PDB ID: 7TT9) as detailed in the supporting information. The asymmetric unit contains four monomers forming the G–H and C–FG interfaces. On the distal side and near the normal to the heme plane through the iron, the electron density map accommodates a single heteroatom, modeled as a water molecule. The Fe–O distance ranges from 2.60 to 2.74 Å in the four chains, longer than 2.1 to 2.3 Å expected for iron coordination (58, 59). Elongated Fe–O distances have been observed in variants of *Methanosarcina acetivorans* protoglobin (60). Like Y34F S2SbHbN, the distal cavity of these proteins does not contain residues capable of directly stabilizing the ligand with hydrogen bonds. In the case of Y34F S2SbHbN in solution, a 5c species is not consistent with the observed electronic absorption features (Figs. 3 and S2) or the NMR data (Fig. S4), and a 6c, aquomet interpretation is preferred. Elongated Fe–O bonds in crystallographic data may be attributable to X-ray-induced photoreduction in the crystal (61).

The structure of cyanomet Y34F S2SbHbN, refined to 1.35 Å (PDB ID: 8VIJ), showed the cyanide ligand to be practically on the normal to the heme plane and not within hydrogen bonding distance of any atoms. Except for the Y34F substitution and the rotameric states of some surface residues, the crystallographic tetramers of S2SbHbN (PDB ID: 8UGZ) and Y34F S2SbHbN (PDB ID: 7TT9) are superimposable within data resolution (RMSD of 0.1 Å over 116 CA pairs). The rings of Phe(CD1)34 and Tyr(CD1)34 occupy the same position, pointing to the heme CHD atom (β meso carbon). Thus, the reactivity difference between the two versions of the protein appears solely linked to the presence of the tyrosine hydroxyl group.

### Self-association of S2SbHbN in solution

**Figure 8:**
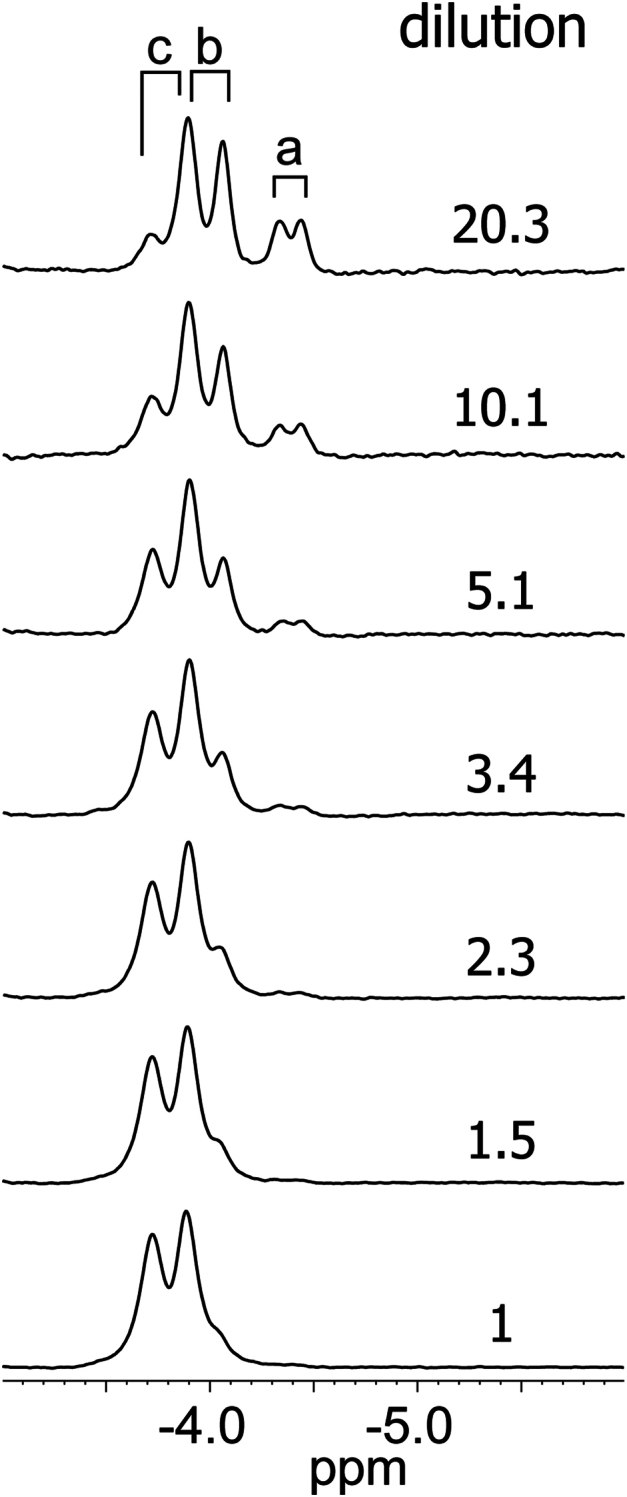
**1D**^**1**^**H NMR spectra of cyanomet S2SbHbN at variable concentration (600 MHz and 25 °C).** The initial high-concentration (∼5 mM) sample (*bottom trace*) was diluted stepwise to low concentration with additional sample buffer of 20 mM potassium phosphate, pH ∼7.5, 90% ^1^H_2_O/10% ^2^H_2_O while maintaining a twofold excess of KCN. The selected resonances are from the *β* protons of a heme vinyl, established with two-dimensional homonuclear data (Fig. S9). The full spectra are shown in Fig. S8. Peaks labeled a, b, and c are from different conformations or association states; a is assigned to a monomeric state. Dissociation constants are in the high micromolar range. S2SbHbN, *Shewanella benthica* strain KT99 TrHbN-2 (UniProt ID: A9DF82) with Cys51Ser and Cys71Ser replacements.

Solution NMR data were collected to enhance the information provided by the X-ray models of S2SbHbN and test the applicability of PISA results. Even when excess KCN is added to the ferric protein to yield the low-spin cyanomet state, the proton NMR spectrum shows lines broader than typically obtained for monomeric TrHbNs (Fig. S8) in a likely manifestation of conformational fluctuations and oligomerization. These processes are evident in the multiplicity of lines connected by exchange cross peaks in NOESY and TOCSY data (Fig. S9). Line intensity (Figs. 8 and S8) and rotational correlation time (62) (Fig. S10) show a strong protein concentration dependence in the micromolar to millimolar range. In this multistate situation, a rigorous extraction of dissociation constants was not feasible. However, the specificity of self-association was probed by mutagenesis targeting residues across the G–H interface in the crystal.

### The structure of G–H interface variants

**Figure 9:**
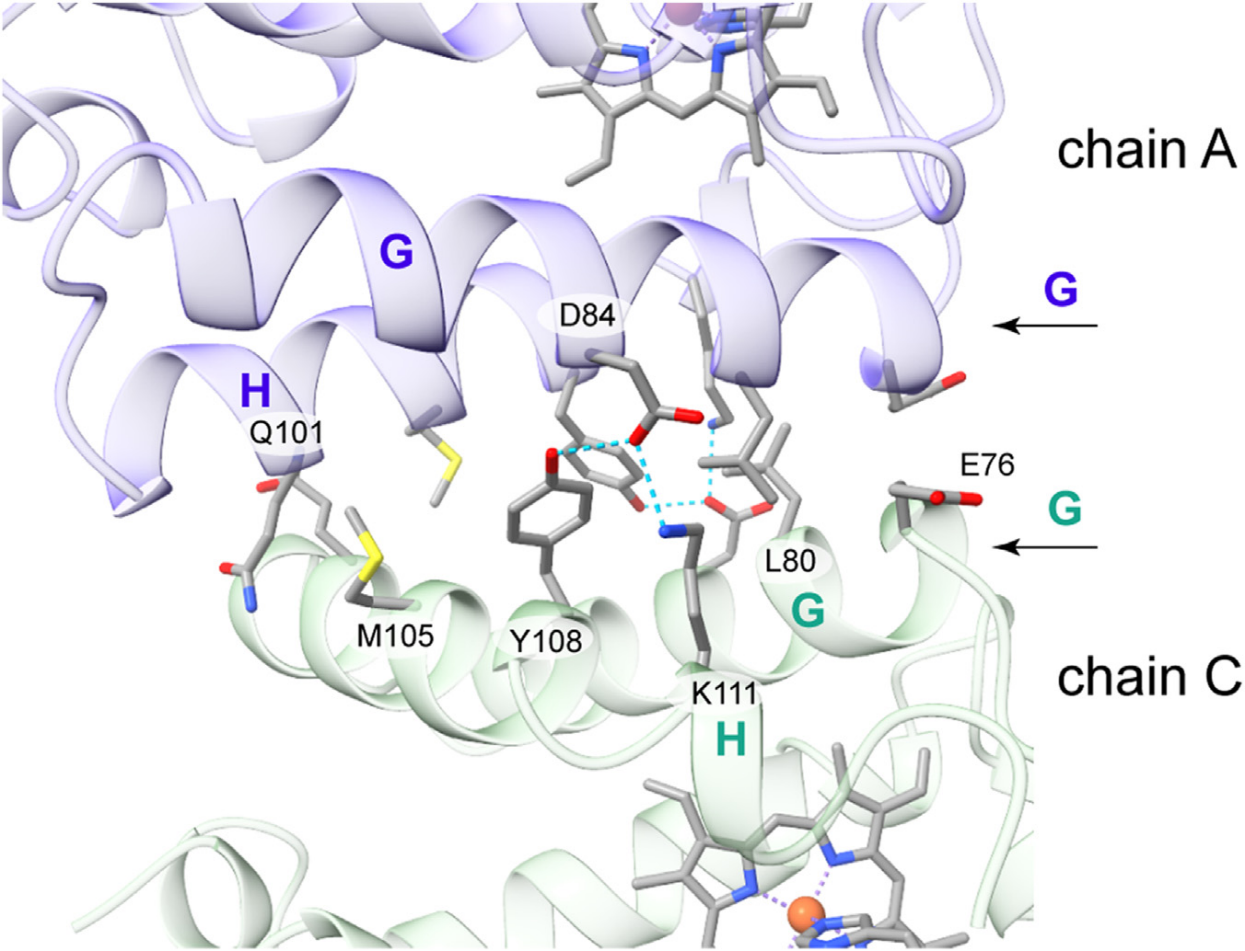
**The G–H interface observed in crystals of cyanomet S2SbHbN (Protein Data Bank ID:****8UGZ****) illustrated with chains A and C.** Following the topological labels assigned by Bustamante *et al.* (37), positions are H10 for Tyr108 and H15 for Lys(H15)111 because of variability in the H helix and a two-residue deletion compared with the reference sequence used by these authors. S2SbHbN, *Shewanella benthica* strain KT99 TrHbN-2 (UniProt ID: A9DF82) with Cys51Ser and Cys71Ser replacements.

Figure 9 shows the G–H interface found in the structure of S2SbHbN and Y34F S2SbHbN. The side chain of Tyr(H10)108 in subunit A(C) is within hydrogen bond distance of the side chain of Asp(G11)84 in subunit C(A) and likewise in subunits B and D. The turn of H helix following Tyr(H10)108 presents Lys(H15)111 A(C) to Asp(G10)84 C(A), completing a three-residue H-bond network. Breaking the Tyr(H10)108(A)–Asp(G10)84(C) interaction was expected to destabilize the G–H dimer if present in solution. The programs BeAtMuSIC (63) and MutaBind2 (64) both anticipated that an alanine at position H10 would destabilize the interface significantly. The Y108A substitution was therefore deemed appropriate not only because of the helical propensity of alanine but also because Ala(H10) is the TrHbN-2 consensus residue (Fig. 1).

Y108A S2SbHbN with bound cyanide crystallized in space group P42_1_2. Instead of four subunits, the asymmetric unit contains only two molecules that form essentially the C–FG contacts observed in the pseudo wildtype protein. The structural model, refined to 1.7 Å resolution (PDB ID: 8TLS), has the same secondary and tertiary features as the parent protein. The disruption of the interchain contacts of Tyr(H10)108 and Lys(H15)111 with Asp84 allows Lys(H15)111 to form an intramolecular salt bridge with Glu(G2)76 and changes slightly the relative position of the G and H helices (Fig. S11). This result implies that the residue at position H10 is important for the formation of the dimeric G–H interface. In addition, PISA analysis of the Y108A S2SbHbN structure predicts the variant to be monomeric and suggests that the C–FG interface plays no role in the assembly of S2SbHbN in solution.

Across the G–H interface of the pseudo wildtype protein, the G helix axes are positioned such that Leu(G6)80 from two facing chains are close to each other. The G6 site was also identified by PISA as central to the stability of the dimer. We chose to replace Leu(G6)80 with an alanine to avoid disrupting the helix and to weaken the interactions between chains according to both BeAtMuSIC and MutaBind2. L80A S2SbHbN crystallized in the same space group as the parent S2SbHbN, P2_1_2_1_2_1_. The model, built to 1.9 Å (PDB ID: 8UZU), has four chains per asymmetric unit in the arrangement featuring the C–FG and G–H interfaces. The portion of the G helix encompassing Ala(G6)80 is well defined, but the replacement has a subtle effect on subunit packing. The small side chain at position 80 allows Lys(H15)111 to reorient and form the intramolecular contact observed with Glu(G2)76 in Y108A S2SbHbN (Fig. S11); furthermore, there is no clear electron density for Tyr(H10)108, which seems to be delocalized within the larger available volume. The PISA assessment of the variant suggests a weakened dimer. Evidence for the perturbation of the assembly is provided in the supporting information by one-dimensional NMR spectra of the Y108A and L80A variants in the cyanomet state (Fig. S12).

To probe the role of Lys(H15)111, we prepared the K111I variant, predicted to stabilize the dimer according to BeAtMuSIC and destabilize it according to MutaBind2. To date, we have not been able to obtain crystals of K111I S2SbHbN. Compared with proteins containing Tyr(CD1)34, K111I S2SbHbN has an electronic absorption spectrum (Fig. S2) that indicates further bias to the aquomet state at neutral pH. NMR data (Fig. S4) concur. A possible conduit for the influence of position H15 on heme ligation is by interaction with the adjacent Tyr(G5)79, itself in contact with the heme 2-vinyl and 3-CH_3_ substituents (Fig. S13). In addition, whereas the NMR spectra of L80A and Y108A S2SbHbN in the cyanomet state are simpler than the pseudo wildtype spectrum (*i.e.*, contain fewer and sharper hyperfine-shifted resonances), cyanomet K111I S2SbHbN exhibits partial-intensity broad lines (Fig. S12), supporting slow chemical exchange among multiple species.

### Pressure response of S2SbHbN and variants in solution

**Figure 10:**
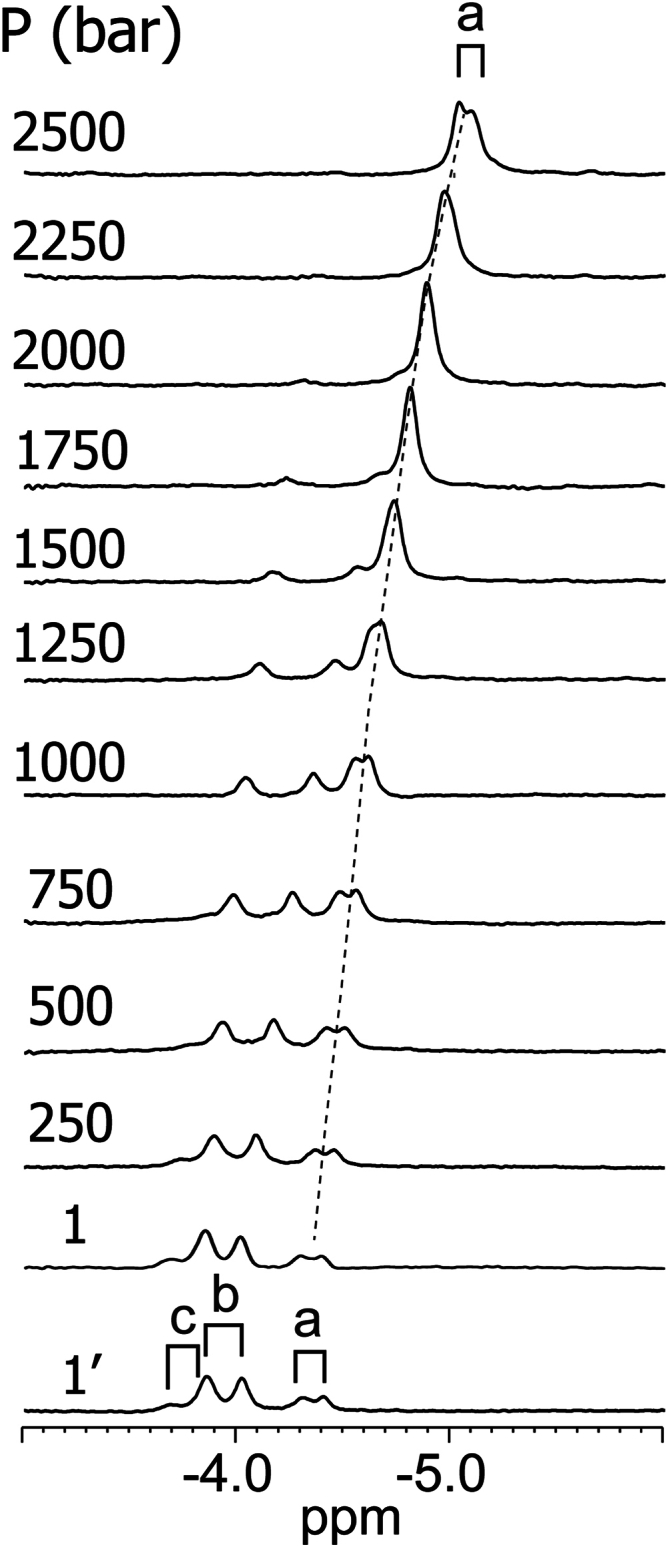
**1D**^**1**^**H NMR spectra of uniformly**^**15**^**N-labeled cyanomet S2SbHbN at variable hydrostatic pressure (∼0.8 mM in 14.12 mM Tris–HCl, 5.88 mM potassium phosphate, pH 7.55, 4 mM KCN, and 90%**^**1**^**H**_**2**_**O/10%**^**2**^**H**_**2**_**O; 600 MHz and 25 °C).** The same heme resonances as in Figure 8 are shown. The bottom-most spectrum (1′) was collected after return from 2.5 kbar to atmospheric pressure to verify reversibility. Labels are as in Figure 8. The downfield regions are shown in Fig. S13. The behavior is consistent with dissociation as pressure increases. S2SbHbN, *Shewanella benthica* strain KT99 TrHbN-2 (UniProt ID: A9DF82) with Cys51Ser and Cys71Ser replacements.

Conformational shifts caused by pressure are associated with volumetric changes through (∂ΔG/∂p)_T_ = ΔV, where ΔG is the Gibbs energy change, ΔV is the molar volume change, and p is pressure. Denaturation of globular protein by pressure has been attributed to packing defects inaccessible to water molecules in the folded state (65). SbHbN is highly porous (Fig. 6) and has packing defects throughout the structure (Fig. S6), which raises the question of its resistance to pressure. We subjected a sample of uniformly ^15^N-labeled cyanomet S2SbHbN to increased hydrostatic pressure and monitored the response of the ^1^H spectrum and ^15^N–^1^H heteronuclear single quantum coherence cross peaks. Figures 10, S14–S16 show that the spectra became less crowded as the pressure was raised, presumably because dissociation (66, 67) eliminated high molecular weight signals while consolidating those of the monomer. It is interesting that one set of β-vinyl protons persists at 2 kbar with coincident chemical shifts as observed for the Y108A and L80A proteins at atmospheric pressure. The experiment demonstrates that the monomeric structure remains highly organized and retains the heme group up to the highest pressure tested.

**Figure 11:**
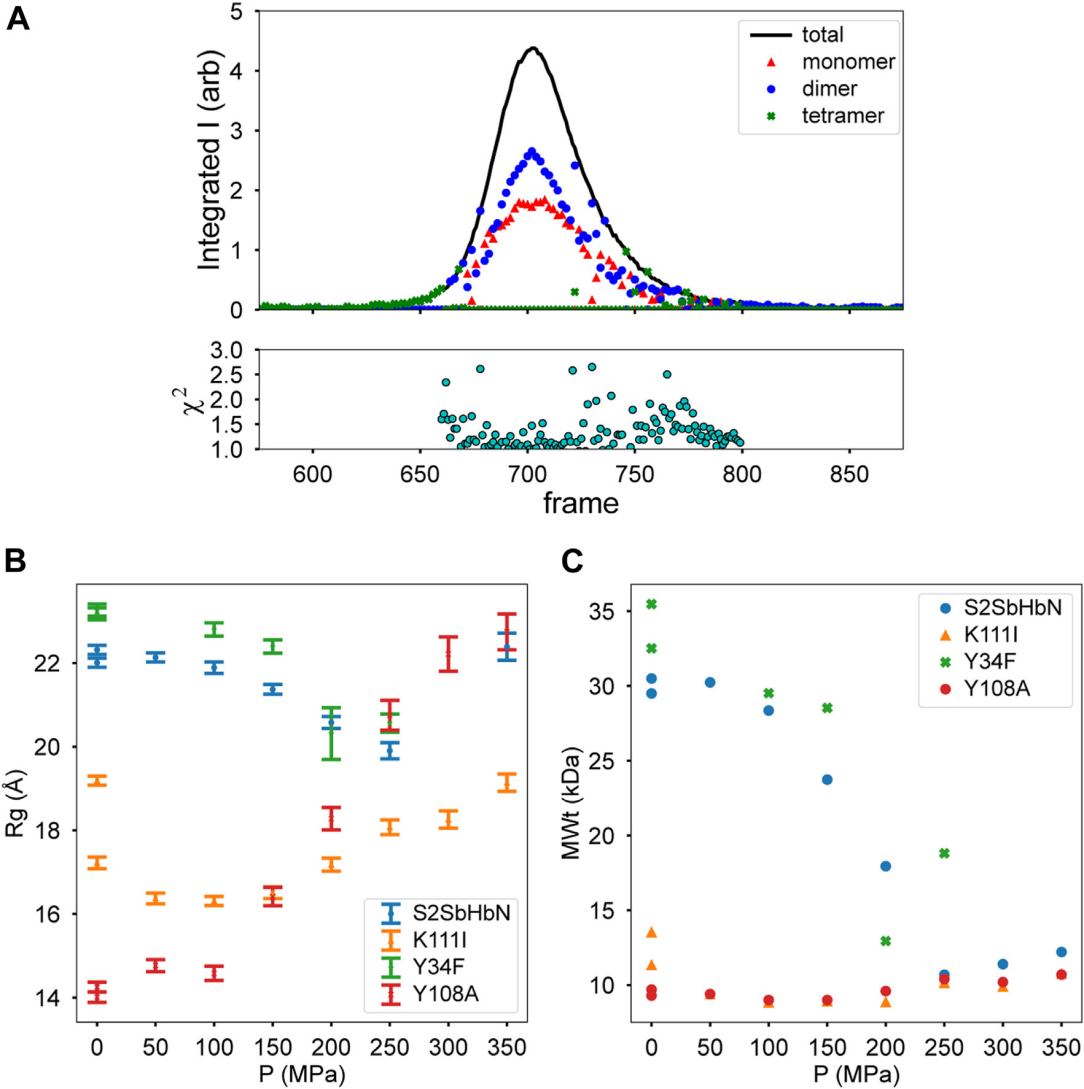
**SAXS and HP-SAXS data on S2SbHbN and variants.***A*, ambient pressure SEC–SAXS data collected on S2SbHbN (∼150 μM heme, pH 7). The integrated intensity (arbitrary units) is plotted *versus* frame number. Based on calculated (FoXS) scattering profiles of monomer, dimer, and tetramer models constructed from Protein Data Bank ID 7TT9, volume fractions of the oligomers are plotted as *symbols* to represent the fraction of the various oligomers contributing to the elution profile. Goodness-of-fit χ^2^ is plotted below. At this sample concentration (∼2 mg/ml at peak), no tetramer is observed, and the dimer–monomer ratio shows strong concentration dependence favoring dimer at peak. *B* and *C*, the pressure response of selected variants represented as the radius of gyration (Rg, Å) and molecular weight (MWt, kDa). S2SbHbN and Y34F variants dissociate to lower oligomeric states above 150 MPa, whereas K111I and Y108A variants remain monomeric at all pressures. HP-SAXS, high-pressure SAXS; SAXS, small-angle X-ray scattering; SEC, size-exclusion chromatography; S2SbHbN, *Shewanella benthica* strain KT99 TrHbN-2 (UniProt ID: A9DF82) with Cys51Ser and Cys71Ser replacements.

Typical protein concentrations for these high-pressure (HP) NMR experiments were in the high micromolar range and favored complex oligomeric mixtures (Figs. 8 and S8). To characterize self-association as a function of pressure at more dilute concentrations, we performed size-exclusion chromatography–small-angle X-ray scattering (SAXS) and HP SAXS on selected proteins. Figure 11*A* shows the “SAXS chromatogram” of S2SbHbN at atmospheric pressure. Each time point (frame) in the chromatogram represents an X-ray scattering profile of an exposure of 2 s. Using models of the monomer, putative dimer, and tetramer from PDB 7TT9, the total integrated scattering intensity has been decomposed into contributions from each proposed individual species based on computed volume fraction (see the *Experimental procedures*, SAXS section). The tetrameric model is taken as the whole unit cell (chains A, B, C, and D) shown in Figure 7, whereas the dimer was chosen as the pair of monomers that preserves the helix–helix interface: chains A + C.

Near the leading and trailing edges of the elution peak, where the sample is relatively dilute, S2SbHbN exists in solution as a roughly 50:50 mixture of dimer and monomer. On the timescale of the experiment (seconds), individual oligomers are not separated, implying rapid equilibrium. At the peak, where protein concentration is higher (∼150 μM), there is an excess of dimer, indicating concentration-dependent oligomerization. However, there appears to be no evidence of the proposed tetramer under these conditions. The χ^2^ statistic plotted at the bottom of Figure 11*A* is near unity for most of the elution profile, indicating that the monomer–dimer mixtures describe the experimental data to within experimental noise levels of the individual scattering curves.

A second putative dimer exists with nontrivial interfacial contact: chains A + D. When included in the volume fraction calculation along with the chain A + C dimer, the OLIGOMER program (see the *Experimental procedures* [SAXS] section) invariably assigns zero volume fraction to dimer A + D. Fitting the models to the peak profile in the SAXS chromatogram (frame 703), dimer A + C yields a 40:60 mixture of monomer and dimer with χ^2^ = 1.01, whereas dimer A + D yields a 30:70 mixture with χ^2^ = 1.48 (Fig. S17). The SAXS data therefore favor the dimer structure that preserves the helix–helix interface.

As the pressure exceeds 150 MPa, both radius of gyration and estimated molecular mass fall. HP volume–fraction analysis supports the existence of some tetramer in both S2SbHbN and Y34F S2SbHbN at ambient pressure, which dissociates to dimer (26.9 kDa) and then to monomer (13.4 kDa) under pressure (Table S4). K111I and Y108A remain largely monomeric at all pressures although some dimer may be present in K111I. The presence of higher oligomers in the HP measurements is consistent with concentration-dependent oligomerization since the sample concentration is likely much higher than in the SAXS chromatogram.

Even though the maximum cellular concentration of SbHbN is unknown, it is unlikely that the dimer is a functional state. The ensemble of the data also suggests that Y108A S2SbHbN is best suited for further study of the determinants of pressure resistance in a “porous” protein and that the G–H interface is weakened in K111I S2HbHbN. Further details of the HP-SAXS measurements can be found in the *Experimental procedures* (SAXS) section and in Table S5.

Our experimental sampling of G–H variants showed varied results and can be viewed as a test of available docking software. AlphaFold (AF)-Multimer (68) is a recent addition to the arsenal of computational tools for the prediction of protein–protein interfaces. It is noteworthy that AF, asked to produce a tetramer of S2SbHbN subunits, predicts correctly the G–H dimer interface but not the C–FG interface (Fig. S18). Asked to produce a dimer of the Y108A variant, AF fails (Fig. S18), in agreement with experiments. In contrast, G–H dimers of both L80A and K111I variants are predicted with high confidence.

### SbHbN relatives and factors related to hydrostatic pressure resistance

A BLAST search using SbHbN as the query retrieved 21 closely related proteins (multiple sequence alignment in Fig. S19) from *Shewanella* species with varying degrees of piezotolerance. Most of the protein sites discussed previously are fully conserved, including Tyr(CD1), which occurs in fewer than 3% of the ∼1500 TrHbN-2 sequences in the conserved domain database. Variations at G6 (Leu, Met, and Val) and H15 (Lys and Arg) in the G–H interface are not expected to cause a dramatic difference in self-association behavior. Because near millimolar cellular concentrations of TrHb are unlikely, the biologically relevant unit is assumed to be devoid of quaternary structure in all cases. Strict conservation of the residues at B10, B13, CD1, E7, E11, and G8 and high conservation of the residues lining the tunnels shown in Figure 6 suggest that the ligand-binding properties and reactivity of these proteins are similar. The formation of tunnels and cavities comparable to those in S2SbHbN is supported by modeling with AF. Thus, the set of 22 proteins is reasonably expected to share a common function through a common mechanism. The relationship to psychropiezophilic adaptation, however, is unclear.

With the available characterization of SbHbN, it is instructive to ask if distinguishing features of piezophily are apparent at any structural level. In previous studies, we inspected the hydrostatic pressure response of two monomeric TrHbN-1s, GlbN from the mesophilic cyanobacterium *Synechococcus* sp. PCC 7002 and THB1 from the green alga *C. reinhardtii*. Both TrHbN-1s are endogenously hexacoordinate and show no obvious tunnels when the distal histidine (GlbN) or distal lysine (THB1) is ligating the heme iron. Decoordination of the endogenous distal ligand is coupled to a conformational change that creates in each protein a network of intramolecular tunnels similar to that of SbHbN (52, 69). Under pressure, whether the tunnels are present or not, these proteins maintain their fold (70, 71) (Fig. S20). This suggests that the volume of the tunnels does not contribute to ΔV of unfolding; in other words, and following the current view of protein stability, this implies that the apparent tunnels in the structure are freely accessible to solvent. Other packing imperfections may aid in minimizing deformation under pressure (72).

Compositional analysis shows that on average TrHbN-1s and TrHbN-2s have 35% of residues thought to help piezophily (subset of polar, small, hydrophilic (15), *i.e.*, Ala, Gly, His, Asn, Gln, and Arg; Table S6). The percentages for SbHbN, GlbN, and THB1 are, respectively, 32%, 37%, and 41%. Across all proteins in the UniProt database, the representation of the same set of residues is 31%. Inspection of the percentages on a per-sequence basis and of structure-based distribution (*i.e.*, surface and buried) is equally inconclusive (Fig. S21), perhaps indicating that the requirements of the TrHb fold (2-on-2 helical sandwich) and functional necessity (access tunnels) impart resistance to pressure.

## Conclusions

The *S. benthica* Hb described here belongs to the TrHbN-2 clade of truncated globins. To our knowledge, SbHbN is the first example of TrHb to be studied from that clade and also the first from a pressure-resistant organism. As expected, the fold bears a strong resemblance to other TrHbNs in helix lengths, helix caps, flexibility of interhelical regions, and overall three-dimensional shape. The sequence of SbHbN differs from the TrHbN-2 consensus sequence at several positions recognized as critical to function. We focused on position CD1 and noted that the hydroxyl group of the wildtype tyrosine influences dioxygen binding. Added to the role of Tyr(CD1) in the irreversible modification of heme and protein by peroxides (33), the observation hints at possible cellular involvement in redox balance and reactive oxygen species detoxification. Amino acid sequence comparisons suggest that these features are shared with *Shewanella* species with different optimal growth conditions and therefore cannot be uniquely associated with life in the cold and deep oceans. Further amino acid composition analyses of TrHbNs yields little insight into piezotolerance. Although we observed that SbHbN has a propensity to self-associate through interactions traced to the G and H helices, because the estimated dissociation constant is in the high micromolar range at 1 atm and increases with hydrostatic pressure, self-assembly is not expected to be physiologically relevant. In contrast, the fold of SbHbN and its ability to retain the heme group are maintained beyond 1 kbar. Interestingly, these features are not specific to this piezophile’s globin as they are observed of TrHbN-1s from organisms suited to atmospheric pressure. The similarity between SbHbN and nonpiezophilic TrHbs suggests that the functional resilience of heme cavity and tunnels within the monomeric structure is related to the ancient origin of the TrHb helical structure. It is conceivable that this tertiary fold developed under extreme conditions early in evolution, making pressure resistance an inescapable byproduct that alleviates the detrimental effects of compression on the protein conformation in extant TrHbs. Further comparative studies of TrHbs from related species thriving under diverse pressure and temperature profiles is expected to clarify the evolutionary origins of these modern protein structures and their functions.

## Experimental procedures

### Protein preparation

The pseudo wildtype protein (C51S/C71S SbHbN or S2SbHbN) and variants (Y34F, L80A, Y108A, and K111I) in the S2SbHbN background were prepared as previously reported (33). Proteins were purified from inclusion bodies except K111I S2SbHbN, which was found mostly in the soluble fraction and obtained with a modified protocol (73). S2SbHbN was labeled uniformly with ^15^N by supplying ^15^NH_4_Cl as the sole nitrogen source in M9 growth medium. Reconstitution of the holoprotein from apoprotein and ferric heme was achieved as reported (44). Saturation was reached at a Soret-to-280 nm ratio dependent on the variant and ranging between 5.1 and 7.8. Sodium dodecyl sulfate polyacrylamide gel electrophoresis confirmed the purity of the proteins. Pure holoprotein solutions were buffer-exchanged with an Amicon (MilliporeSigma) ultrafiltration cell to obtain a final concentration of 5 mM potassium phosphate, pH 7.0. When necessary, the solutions were lyophilized, and the dried proteins were stored at −20 °C for later use.

Molecular masses obtained by intact protein ultraperformance liquid chromatography–mass spectrometry (Acquity/Xevo-G2 system) demonstrated the cleavage of the initial methionine and were consistent with the intended amino acid sequences. Ultraperformance liquid chromatography–mass spectrometry data were processed with BiopharmaLynx.

### UV–visible spectroscopy

To determine protein concentrations, the holoproteins (pseudo wildtype and variants) were subjected to the hemochromogen assay (74, 75) in triplicates. Samples were prepared in potassium phosphate buffer, pH 7, with 5 to 10 μM protein in quartz cuvettes of 1 cm. Data were collected at room temperature in the 250 to 750 nm range on a Varian Cary50 spectrophotometer (Agilent). Tables S1 and S2 list extinction coefficients and absorption maxima.

The response of the ferric form of (Y34F) S2SbHbN to solution pH was monitored as previously described (69) by adding increasing amounts of base (NaOH) or acid (HCl) to samples originally at pH 7. Binding of cyanide was inspected in the ferric form by addition of excess KCN (10 mM). Anoxic reduction to the ferrous form was performed by addition of 2 mM sodium dithionite and equilibration until no spectral changes were observed. Enzymatic reduction and association with dioxygen were observed using a ferredoxin (Fd)–NADPH reductase system (51); the reaction mixture contained 0.04 mg/ml bovine catalase, 2.5 μM spinach Fd, 25 mU spinach Fd–NADP^+^ reductase, and 300 μM NADPH (all enzymes and reagents obtained from Sigma).

### X-ray structure determination

We describe the approach for Y34F S2SbHbN, which was solved first for reasons of chemical stability (33). A ∼15 mg/ml solution of ferric Y34F S2SbHbN was initially incubated with excess KCN and combined in a 1:1 ratio with reservoir solution consisting of 0.1 M Bis–Tris, pH 6.5, and 28% w/v polyethylene glycol monomethyl ether 2000. Crystals were obtained at room temperature *via* the hanging drop vapor diffusion method. For data acquisition, promising crystals were suspended in nylon cryoloops mounted on CrystalCap copper magnetic ported bases (Hampton Research) and flash-cooled in liquid nitrogen for screening. A diffraction dataset on one such crystal was collected to 2.0 Å resolution using Cu K*α* radiation on a Rigaku Oxford Diffraction Supernova diffractometer, processed, and scaled using CrysAlis^Pro^ software (Agilent).

Experimental single-wavelength anomalous diffraction phasing was performed using PHENIX AutoSol (76), which identified four heavy atom (Fe) sites per asymmetric unit. This step was followed by density modification and automated model building with an initial molecular replacement solution obtained with the structure of *Tetrahymena pyriformis* TrHbN in the ferric form (Y25F variant, PDB ID: 3AQ7, (77)). Several rounds of iterative manual model building in Coot software (78) and automatic refinement using phenix.refine were performed, followed by model quality assessment with MolProbity (79). Coordination of the NE2 atom of the proximal histidine (His69) to the heme iron was applied as a restraint. Interactive visualization and analysis were performed with UCSF ChimeraX (80). Internal cavities and tunnels were identified with MOLEonline, version 2.5 (81). Secondary structure was identified with DSSP (82) as implemented in ChimeraX (80).

The protocols for S2SbHbN and its other variants were similar and are included in the supporting information. For these other structures, we relied on single-wavelength anomalous diffraction to obtain experimental phases using a Hybrid Structure Search (83) and Phaser (84) in the Phenix suite (76). When the electron density maps were consistent with cyanide as a distal ligand, a Fe–CN distance restraint was applied. Table S3 contains all crystallographic data and refinement statistics.

### NMR spectroscopy

NMR samples were prepared from lyophilized ferric proteins resuspended to the desired concentrations. As needed, cyanide was added in excess to form the cyanomet complex. All data were obtained on a Bruker Avance II 600 MHz with a triple-resonance cryoprobe (Bruker). Chemical shifts were directly and indirectly referenced to the ^1^H_2_O signal (4.77 ppm at 25 °C). [^15^N,^1^H]-TRACT experiments (62) followed a standard protocol (85). HP NMR experiments were performed in a baroresistant buffer as previously described (71), using a zirconia HP-NMR tube and an Xtreme-60 pump (Daedalus Innovations). At each 250-bar (25 MPa) step from ambient pressure to 2.5 kbar (250 MPa), the sample was allowed to equilibrate for at least 15 min prior to NMR parameter optimization and data collection. Chemical shift calibration used the published pressure dependence (86). Spectral changes with increasing pressure were fully reversible. NMR data were analyzed in TopSpin (Bruker), NMRPipe (87), and Sparky (88).

### Small-angle X-ray scattering

Samples were spun at 14,000 RPM for 10 min prior to measurement to remove dust, bubbles, and aggregates. A 100 μl injection volume of S2SbHbN sample at 10 mg/ml (∼700 μM monomer) was run on a Superdex 200 Increase 10/300 column (Cytiva) equilibrated with 14 mM Tris buffer, pH (∼7) containing 6 mM potassium phosphate. SAXS scattering profiles were collected continuously (every 2 s) from eluted sample flowing at 0.5 ml/min. Based on previous experiments, an approximate fivefold dilution of sample is expected because of the column configuration and X-ray flow cell. Incident X-ray beam flux was 1.22 × 10^12^ photons/s at 9.99 keV. Each image was normalized by transmitted beam intensity. The EIGER 4M detector images were processed using the BioXTAS RAW software (89) to calculate radius of gyration and estimated molecular mass. Additional details are listed in Table S5.

Monomer and putative dimer and tetramer models were constructed from Y34F S2SbHbN (PDB ID: 7TT9) and used as input to the OLIGOMER program in ATSAS, version 3.0.2 (90). Calculations were confined to a range between *q*_*min*_ = 0.013 and *q*_*max*_ = 0.2 with parasitic background corrected using the “-cst” software option.

HP SAXS was performed at CHESS S7A beamline using the apparatus and protocol previously described (91, 92). Plastic disposable sample cells with polyimide windows 7 μm thick were loaded with ∼60 μl of undiluted sample (10 mg/ml) or buffer and sealed, bubble-free, with silicon-based vacuum grease. The cells were submerged in the pressurizing medium (water) and sealed in the HP apparatus. Care was taken to use profiles collected from the same plastic cell when subtracting buffer background from sample. The 14.04 keV X-ray beam was attenuated to 2.1 × 10^11^ photons/s, and wait periods of 5 min were inserted between 4 × 1-s exposures to manage radiation damage. Damage was assessed by applying the CORMAP algorithm (93) to the successive four exposures at each pressure. Pressure was incremented in steps of 100 MPa starting at 0 MPa. A final 0 MPa profile was compared with the initial 0 MPa profile to access cumulative damage. Additional pressure points were taken at 100 MPa intervals starting at 50 MPa, to fill out the pressure series. Additional details for the HP-SAXS experiment can also be found in Table S5.

## Data availability

Relevant structures have been deposited with the PDB: 7TT9, Y34F S2SbHbN; 8VIJ, cyanomet Y34F S2SbHbN; 8TLS, cyanomet Y80A S2SbHbN; and 8UZU, cyanomet L80A S2SbHbN. Relevant SAXS data have been deposited with the Small Angle Scattering Biological Data Bank: SASDWK4, S2SbHbN; SASDWL4, S2SbHbN 0.101 MPa; SASDWM4, S2SbHbN 0.101 MPa (final measurement after end of pressure series); SASDWN4, S2SbHbN 50 MPa; SASDWP4, S2SbHbN 100 MPa; SASDWQ4, S2SbHbN 150 MPa; SASDWR4, S2SbHbN 200 MPa; SASDWS4, S2SbHbN 250 MPa; SASDWT4, S2SbHbN 350 MPa; SASDWU4, K111I S2SbHbN 0.101 MPa; SASDWV4, K111I S2SbHbN 0.101 MPa (final measurement after end of pressure series); SASDWW4, K111I S2SbHbN 50 MPa; SASDWX4, K111I S2SbHbN 100 MPa; SASDWY4, K111I S2SbHbN 150 MPa; SASDWZ4, K111I S2SbHbN 200 MPa; SASDW25, K111I S2SbHbN 250 MPa; SASDW35, K111I S2SbHbN 300 MPa; SASDW45, K111I S2SbHbN 350 MPa; SASDW55, Y34F S2SbHbN 0.101 MPa; SASDW65, Y34F S2SbHbN 100 MPa; SASDW75, Y34F S2SbHbN 150 MPa; SASDW85, Y34F S2SbHbN 200 MPa; SASDW95, Y34F S2SbHbN 250 MPa; SASDWA5, Y108A S2SbHbN 0.101 MPa; SASDWB5, Y108A S2SbHbN 0.101 MPa (replicate); SASDWC5, Y108A S2SbHbN 50 MPa; SASDWD5, Y108A S2SbHbN 100 MPa; SASDWE5, Y108A S2SbHbN 150 MPa; SASDWF5, Y108A S2SbHbN 200 MPa; SASDWG5, Y108A S2SbHbN 250 MPa; SASDWH5, Y108A S2SbHbN 300 MPa; and SASDWJ5, Y108A S2SbHbN 350 MPa.

## Supporting information

This article contains supporting information (15, 33, 38, 44, 54, 62, 68, 71, 74, 75, 80, 95, 96, 97, 98, 99, 100, 101, 102, 103, 104, 105, 106, 107, 108, 109, 110, 111, 112, 113, 114, 115, 116, 117, 118).

## Conflict of interest

The authors declare that they have no conflicts of interest with the contents of this article.
