# Supplementary material for: Extremophilic hemoglobins: The structure of Shewanella benthica truncated hemoglobin N
Source: J Biol Chem. 2025 Jan 24;301(3):108223. doi: 10.1016/j.jbc.2025.108223 (PMC11904497; doi:10.1016/j.jbc.2025.108223)
Supplement: Supporting information [file mmc1.pdf]

Supporting Information

Extremophilic hemoglobins: The structure of *Shewanella benthica* truncated hemoglobin N

Jaime E. Martinez Grundman, Thomas D. Schultz, Jamie L. Schlessman, Eric A. Johnson, Richard E. Gillilan, Juliette T.J. Lecomte

Table of Content

Structural alignment procedure		S-2
Structural features of S2SbHbN		
Crystallization and structural notes for Y34F S2SbHbN		S-2
Table S1	Extinction coefficients used for concentration determination	S-4
Table S2	Wavelength (nm) of electronic absorption maxima	S-4
Table S3	Data collection and refinement statistics for the crystal structure of S2SbHbN variants	S-5
Table S4	Oligomeric volume fractions under pressure	S-6
Table S5	SAXS data collection parameters	S-7
Table S6	Amino acid composition of TrHbNs	S-8
Figure S1	Phylogenetic tree of TrHbNs	S-9
Figure S2	Electronic absorption spectra of S2SbHbN and variants	S-10
Figure S3	pH titration of S2SbHbN and Y34F S2SbHbN	S-11
Figure S4	Downfield hyperfine shifted region of the ^{1}H NMR spectra of ferric S2SbHbN and variants	S-12
Figure S5	Electronic absorption spectra of S2SbHbN in the presence of cyanide	S-13
Figure S6	Cavities in the structure of cyanomet S2SbHbN	S-14
Figure S7	$2mF_o{-}DF_c$ electron density contoured at 1.0 σ showing where the four chains meet in the asymmetric unit of cyanomet S2SbHbN	S-15
Figure S8	1D ^{1}H NMR spectra of cyanomet S2SbHbN at variable protein concentration	S-16
Figure S9	Homonuclear NOESY and TOCSY data for heme vinyl β protons	S-17
Figure S10	1D [^{15}N,^{1}H]-TRACT data of cyanomet S2SbHbN	S-18
Figure S11	Interaction of Lys111 with Glu76 and interhelical polar plot	S-19
Figure S12	^{1}H NMR spectra of cyanomet S2SbHbN and variants	S-20
Figure S13	Proximity of Ile(H15)111, Tyr(G5)79 and the heme group in cyanomet K111I S2SbHbN	S-21
Figure S14	Downfield region of 1D ^{1}H NMR spectra of ^{15}N-labeled cyanomet S2SbHbN at variable hydrostatic pressure	S-22
Figure S15	^{15}N-^{1}H HSQC spectra of ^{15}N-labeled cyanomet S2SbHbN at variable hydrostatic pressure	S-23
Figure S16	Region of the spectra in Figure S15	S-24
Figure S17	Comparison of scattering profiles for two candidate dimer structures	S-25
Figure S18	AlphaFold-Multimer results for S2SbHbN and variants	S-26
Figure S19	Alignment of the twenty-one *Shewanella* HbN sequences	S-28
Figure S20	^{15}N-^{1}H HSQC spectrum of aquomet THB1 at 2.5 kbar	S-30
Figure S21	Amino acid composition of TrHbNs	S-31
References		S-33

Structural alignment procedure

A total of 12,745 TrHbN sequences were retrieved from the NCBI Conserved Domain Database (January 2021, accession code cd00454). Redundant sequences (100% identical), unnatural sequences derived from PDB, or sequences shorter than 100 amino acids were removed using JalView (95). Some of the remaining 5,511 sequences contained multiple domains or partial reads. The set was processed further with in-house scripts using command-line BLAST+ (96) to identify and extract individual TrHbN domains within each sequence. Extracted domain sequences shorter than 100 amino acids or displaying 90% identity with another sequence were removed (3,024 remaining). A multiple sequence alignment was performed with PROMALS3D (97) (default settings and homology information from the following TrHbN PDB coordinates: 1DLY, 1DLW, 1RTE, 5AB8, 3AQ6, 1RTX, 1S69, 2HZ2, 4MAX, 4L2M, 4XDI, 6CII, 6BME, 6TD7). Sequence gaps and insertions were trimmed using the Block Mapping and Gathering with Entropy (BMGE) program available on the NGPhylogeny.fr online server (98, 99) and with the following options: BLOSUM-62 setting, maximum entropy threshold of 0.9, gap rate cut-off of 0.2, and minimum block size of 1. The final MSA was the basis for a maximum likelihood (ML) phylogeny using PhylML 3.3 (100, 101) with the WAG substitution model, an optimized BioNJ starting tree, SH-like branch supports, estimated proportion of invariable sites, four substitution rate categories, and an estimated gamma distribution parameter. The final tree was analyzed using the Interactive Tree of Life (iTOL) online tool (102, 103). In the unrooted tree format, two branches were observed of approximately equal size (Figure S1), which upon further inspection corresponded to the two subgroups described previously (38).

Summary of helix capping and heme propionate interactions in S2SbHbN structure 8UGZ

Notable interactions fastening the secondary structure: Ser(A6)2 OG and the backbone N of Glu(A9)5 (N-cap to A helix); Thr(B15)26 OG1 and the backbone O of Asp(B11)22 and Thr(B12)23 (C-cap to B helix, securing a 3_{10} turn); Asp(E3)38 OD1 and the backbone N of Arg(E6)41 (N-cap to E helix); Ser(F3)64 and Glu(F6)67 (reciprocal side-chain/main-chain, N-cap to the single-turn F helix); and Asn(G1)75 OD1 and the backbone N of Glu(G4)78 (N-cap to G helix). Of these, the F3–F6 and G1–G4 interactions are different from those structure 2GL3 shown in Figure 4 although the same topology is achieved.

In S2SbHbN, the 6-propionate (on pyrrole D in the PDB nomenclature) and 7-propionate (on pyrrole A) are well defined by the electron density. The 6-propionate carboxylate is within hydrogen-bonding distance of Ser(E2)37 OG while the 7-propionate carboxylate is within hydrogen-bonding distance of the backbone NH of Lys(F2)63. Both functional groups interact with ordered water molecule(s). The 6-propionate makes fewer contacts with protein atoms than the 7-propionate, but both carboxylate groups are accessible to solvent on the surface of the protein.

Crystallization and structural notes for Y34F S2SbHbN

In preliminary crystallization trials of cyanomet S2SbHbN, we noted that the red crystals gradually turned green (33). Protection from light and increased cyanide concentration prevented the transformation and allowed for the structural determination of the cyanomet protein detailed in the main text (PDB ID 8UGZ). Replacement of Tyr(CD1)34 with a Phe also prevented the greening process. Crystals of Y34F S2SbHbN were obtained with bound cyanide

(red) or having lost cyanide (brown). The structure of the "brown Y34F protein" was solved (PDB ID 7TT9) as well as that of the "red Y34F protein" (PDB ID 8VIJ).

When the two Y34F S2SbHbN structures are overlapped, it appears that the distal water molecule of structure 7TT9 is located near the center of the cyanide ion present in structure 8VIJ. Other ordered water molecules within the heme cavity are at the same locations in both forms of the protein.

Table S1 Extinction coefficients used for concentration determination (ferric state)

Protein	ε (mM^{-1} cm^{-1})	wavelength	pH
S2SbHbN	134 ± 2	407	7.5
Y34F	170 ± 1	405	7.0
Y108A	132 ± 7	408	7.0
L80A	124 ± 6	408	7.0
K111I	141 ± 5	408	7.0

Table S2 Wavelength (nm) of electronic absorption maxima (ferric state, pH 7, Figure S2)

Protein	Soret				CT
S2SbHbN	408		544	582	
Y34F	405	502			633
Y108A	407		543	581	
L80A	407		543	581	
K111I	407	500	542	582	627

CT: charge transfer

Table S3 Data collection and refinement statistics for the crystal structure of S2SbHbN variants

	Y34F	Y34F-CN	L80A-CN	Y108A-CN
PDB ID	7TT9	8VIJ	8UZU	8TLS
Crystallization conditions				
Reservoir solution	28% PEG MME 2000, 0.1 M BIS-TRIS (pH 6.5)	23% PEG MME 2000, 0.1M Bis-Tris (pH 6.5), 10 mM KCN	23% PEG MME 2000, 0.1M Bis-Tris (pH 5.1), 10 mM KCN	27% PEG MME 2000, 0.1M Bis-Tris (pH 5.1), 10 mM KCN
Data collection statistics				
Space group	$P2_12_12_1$	$P2_12_12_1$	$P2_12_12_1$	$P42_12$
Cell dimensions a, b, c (Å)	27.38, 105.05, 151.08	27.46 105.15 151.32	27.16, 105.22, 151.13	66.72, 66.72, 125.02
α, β, γ (°)	90.0, 90.0, 90.0	90.0, 90.0, 90.0	90.0, 90.0, 90.0	90.0, 90.0, 90.0
Wavelength (Å)	1.5418	1.5418	1.5418	1.5418
Temperature (K)	110	100	110	110
Resolution (Å)	24.26–2.00 (2.07–2.00)	18.96–1.35 (1.40–1.35)	22.99–1.90 (1.97–1.90)	24.24–1.70 (1.76–1.70)
Total no. of reflections	172732 (13395)	481556 (25627)	199436 (15035)	335198 (25284)
No. of unique reflections	30686 (3074)	97066 (9662)	35447 (3551)	59860 (5978)
Completeness (%)	99.9 (100.0)	99.0 (98.6)	99.9 (100.0)	99.8 (99.7)
Redundancy	5.6 (4.4)	5.0 (2.7)	5.6 (4.2)	5.6 (4.2)
R-sigma	0.028 (0.100)	0.081 (0.367)	0.041 (0.160)	0.042 (0.213)
Mean $I/\sigma(I)$	31.1 (8.4)	11.02 (3.46)	24.4 (5.5)	24.1 (4.1)
$CC_{1/2}$	0.998	0.998	0.998	0.999
Wilson B factor (Å^2)	12.0	7.8	11.3	7.8
Refinement statistics				
Resolution range (Å)	23.45–2.00 (2.07–2.00)	16.97–1.35 (1.38–1.35)	22.99–1.90 (1.95–1.90)	21.90–1.70 (1.74–1.70)
Completeness (%)	99.1 (98.1)	99.1 (98.5)	98.4 (97.7)	99.7 (99.8)
No. of reflections, working set	30388 (2936)	96962 (6821)	34829 (2443)	31798 (2225)
No. of reflections, test set	1983 (195)	1998 (141)	1968 (139)	1995 (140)
R-work (%)	17.5 (18.4)	0.163 (0.200)	0.195 (0.224)	0.189 (0.200)
R-free (%)	20.3 (24.8)	0.200 (0.220)	0.224 (0.265)	0.214 (0.262)
RMSD bond lengths (Å)	0.004	0.008	0.004	0.008
RMSD bond angles (°)	0.54	0.88	0.63	1.07
No. of non-H atoms	4293	4529	4282	2272
Protein	3588	3912	3731	1868
Ligands	172	180	180	90
Solvent	533	437	371	314
Average B-factor (Å^2)	14.6	11.1	14.6	9.5
Protein	13.5	10.3	14.6	8.5
Ligands	10.2	7.7	8.2	6.4
Solvent	23.2	19.3	17.9	16.6
Ramachandran favored (%)	100.0	100.0	98.5	98.7

Table S4 Oligomeric volume fractions under pressure

sample	oligomer	0 MPa	50 MPa	100 MPa	150 MPa	200 MPa	250 MPa	300 MPa	350 MPa
S2SbHbN	A	0.00	0.00	0.00	0.00	0.00	0.69	0.78	0.74
	AC	0.02	0.28	0.43	0.63	0.64	0.18	0.00	0.00
	AD	0.00	0.00	0.00	0.00	0.26	0.00	0.00	0.00
	ABCD	0.98	0.72	0.57	0.37	0.10	0.10	0.20	0.26
	Chi2	0.71	1.49	0.83	0.78	0.50	0.38	0.43	0.43
K111I	A	0.66	0.76	0.76	0.76	0.77	0.76	0.76	0.77
	AC	0.34	0.24	0.24	0.24	0.19	0.18	0.15	0.11
	AD	0.00	0.00	0.00	0.00	0.00	0.00	0.00	0.00
	ABCD	0.00	0.00	0.00	0.00	0.04	0.06	0.09	0.12
	Chi2	0.57	0.56	0.54	0.45	0.54	0.48	0.44	0.44
Y34F	A	0.00	-	0.00	0.00	0.77	0.0	0.65	-
	AC	0.10	-	0.30	0.35	0.14	0.47	0.00	-
	AD	0.00	-	0.00	0.00	0.00	0.31	0.00	-
	ABCD	0.90	-	0.70	0.65	0.92	0.21	0.34	-
	Chi2	0.87	-	0.67	0.67	0.29	0.49	0.23	-
Y108A	A	0.97	1.00	1.00	-	-	0.83	-	0.77
	AC	0.03	0.00	0.00	-	-	0.00	-	0.00
	AD	0.0	0.00	0.00	-	-	0.00	-	0.00
	ABCD	0.0	0.00	0.00	-	-	0.17	-	0.229
	Chi2	0.94	0.78	0.63	-	-	0.49	-	0.46

Volume fractions of proposed oligomers calculated from high-pressure scattering profiles based on models from PDB ID 7TT9 chains A, B, C, and D. OLIGOMER q range: [0.35, 0.2] $Å^{-1}$ using setting "—cst." Chi2 = goodness of fit.

Table S5 SAXS data collection parameters

**Ambient pressure data collection (SEC-SAXS)**	
Instrument	Sector 7A1 Cornell High Energy Synchrotron Source, EIGER 4M (Dectris) detector
Wavelength, energy	1.240 Å, 9.99 keV
Flux	1.22×10^{12} photons/s
q-measurement range	0.008 – 0.557 $Å^{-1}$
Absolute scaling method	Water 25º C
Basis for normalization	Beamstop diode
Software	BioXTAS RAW 2.1.4<br>OLIGOMER (ATSAS 3.0.3-1)
Method of monitoring radiation damage	Return to baseline, Rg and Molecular Weight
Exposure time, number of exposures	Continuous 2 s
Sample configuration	1.5 OD quartz glass capillary with 10 µm thick walls *in vacuo*; SEC: ÄKTA Pure™ system; Superdex 200 10/300 column 4º C
**High pressure data collection (HP-SAXS)**	
Instrument	Sector 7A1 Cornell High Energy Synchrotron Source, EIGER 4M (Dectris) detector
Wavelength, energy	0.882 Å. 14.05 keV
Flux	2.1×10^{11} photons/s
q-measurement range	0.012 – 0.700 $Å^{-1}$
Absolute scaling method	Water 25.6º C (ambient air pressure only)
Basis for normalization	Beamstop diode
Software	BioXTAS RAW 2.1.4
Method of monitoring radiation damage	Successive exposures with CORMAP statistic
Exposure time, number of exposures	4-10 × 1 s exposures
Sample configuration	60 µl sealed plastic cell with 7-µm thick polyimide windows. Path length 3.5 mm, water pressurizing medium with 0.5 mm diamond windows.

Table S6 Amino acid composition of TrHbNs (globin domain only)

	SbHbN n = 116	GlbN n = 123	THB1 n = 120	SbHbN %	GlbN %	THB1 %	TrHbN-1 % (SD)	TrHbN-2 % (SD)
A	12	13	17	10.3	10.5	14.2	10.6 (4.2)	9.6 (3.0)
C	2	0	0	1.7	0.0	0.0	0.3 (0.5)	1.4 (0.7)
D	9	11	7	7.7	8.9	5.8	7.9 (2.8)	7.3 (1.8)
E	11	7	8	9.4	5.6	6.7	7.0 (2.3)	6.3 (2.2)
F	2	7	7	1.7	5.6	5.8	5.2 (1.6)	3.8 (1.4)
G	9	8	10	7.7	6.5	8.3	7.9 (2.4)	8.0 (1.7)
H	2	4	5	1.7	3.2	4.2	3.9 (1.3)	2.8 (1.4)
I	10	3	4	8.5	2.4	3.3	5.2 (2.1)	5.9 (2.2)
K	7	6	5	6.0	4.8	4.2	4.5 (3.0)	5.0 (2.7)
L	8	12	10	6.8	9.7	8.3	9.4 (2.2)	9.7 (2.1)
M	5	3	5	5.1	3.2	4.2	2.7 (1.2)	3.6 (1.3)
N	4	7	2	3.4	5.6	1.7	2.7 (2.2)	4.1 (1.9)
P	3	1	3	2.6	0.8	2.5	2.9 (1.6)	3.2 (1.3)
Q	4	7	6	3.4	5.6	5.0	4.0 (1.9)	4.3 (2.0)
R	6	7	9	5.1	5.6	7.5	5.5 (2.4)	6.1 (2.6)
S	5	4	6	4.3	3.2	5.0	4.2 (1.9)	4.1 (1.9)
T	6	6	2	5.1	4.8	1.7	4.6 (1.7)	4.8 (1.9)
V	6	14	10	5.1	11.3	8.3	8.1 (2.6)	7.1 (2.0)
W	0	0	0	0.0	0.0	0.0	0.2 (0.6)	0.3 (0.5)
Y	5	3	4	4.3	2.4	3.3	3.2 (1.1)	2.7 (1.3)
pI	4.76	5.25	5.92					
PP	37	46	49	31.6	37.1	40.8	34.5	34.9

Numbers are for SbHbN (UniProt ID A9DF82), *Synechococcus* sp. PCC 7002 GlbN (UniProt ID Q8RT58), and *Chlamydomonas reinhardtii* THB1 (UniProt ID A8JAR4) after initial Met cleavage; n is the sequence length. TrHbN-1 and TrHbN-2 numbers are the statistics for the sets included in the phylogenetic analysis. pI is the isoelectric point calculated on the basis of composition (whole protein) by ProtParam (104). PP groups the amino acids preferentially found in psychropiezophiles according to (15): A, G, H, N, Q, R. UniProtKB statistics tabulated on July 24, 2024 list 31.1% of the same residues in the aggregated sequences. Also see Figure S21.

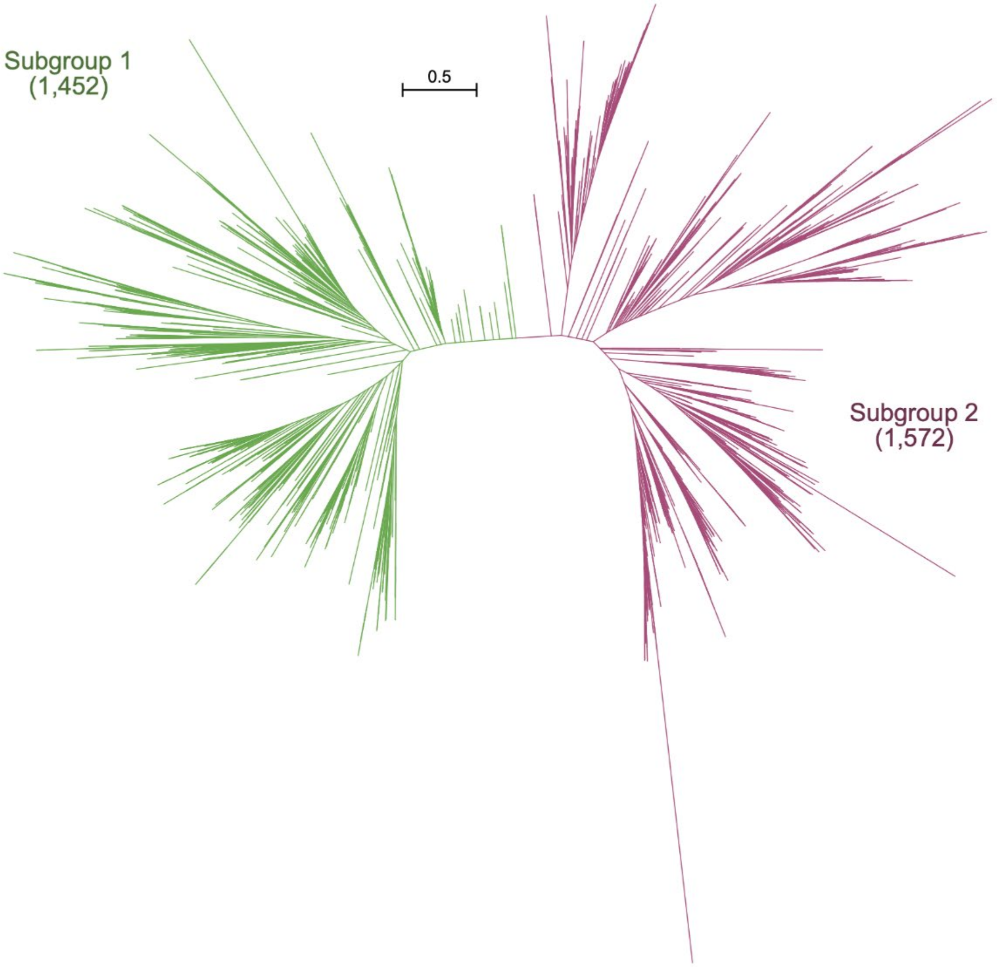

Figure S1 Phylogenetic tree of TrHbNs prepared as described in Section 1 above and illustrating the separation of TrHbN-1 and TrHbN-2 subgroups.

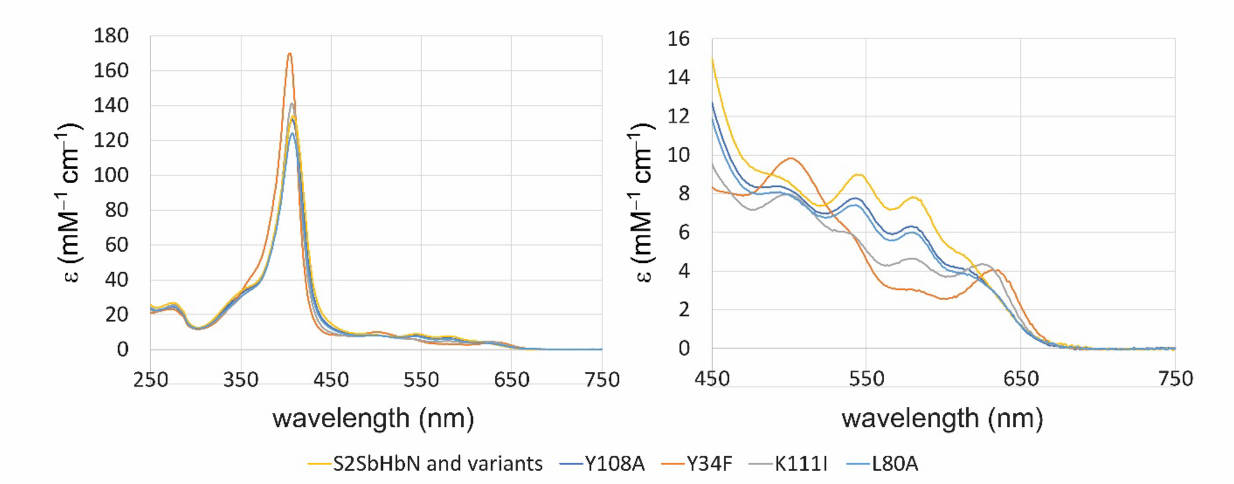

Figure S2 Electronic absorption spectra of proteins discussed in the text (ferric state, pH ~7, room temperature). Data were scaled according to the extinction coefficients obtained by the hemochromogen assay (74, 75) (Table S1). The spectrum of S2SbHbN is consistent with a hydroxymet complex (equilibrium spin 1/2–5/2 mixture). The spectrum of the Y34F variant is consistent with an aquomet complex (spin 5/2). Variants containing Y34 also appear as hydroxymet complexes, except the K111I protein, which favors the high-spin (5/2) state.

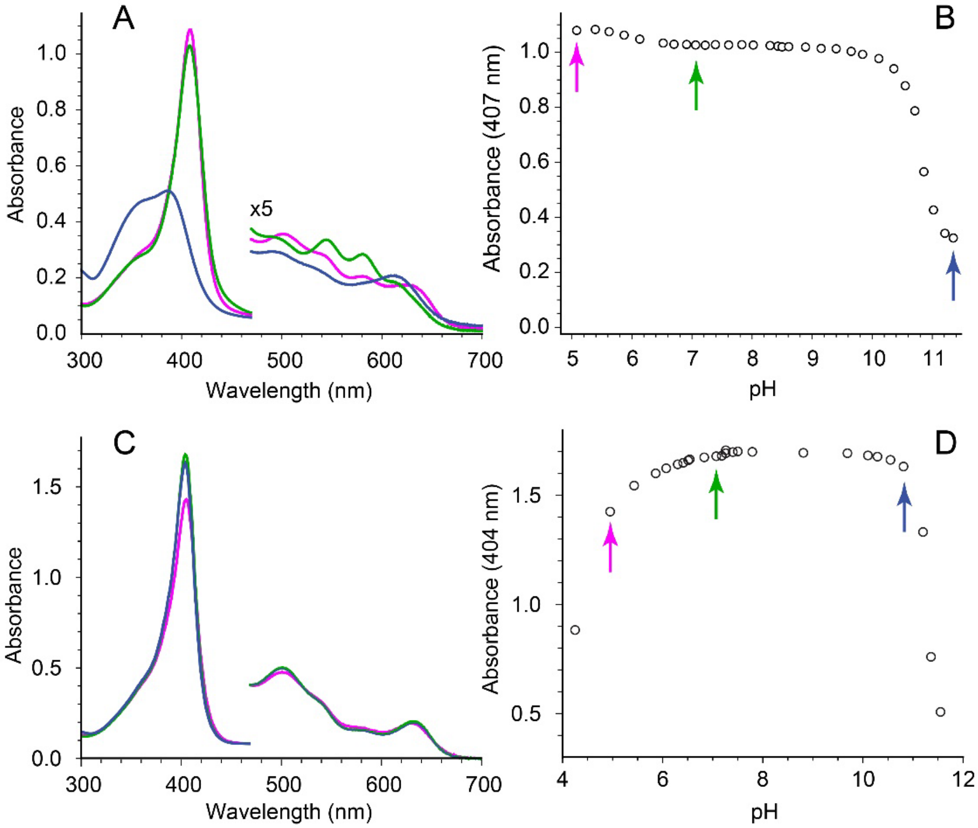

Figure S3 pH titration of S2SbHbN and Y34F S2SbHbN. (A) Reproduced from Figure 2A in the main text, ferric protein at pH 5.1 (magenta), 7.1 (green), and 11.4 (blue). (B) Absorbance at 407 nm as a function of pH. The colored arrows correspond to the pH of the spectra in (A). As the pH is decreased from neutral, the bound hydroxide ion is either protonated to become a bound water molecule (aquomet state), or protonated and released to generate a five-coordinate (5c) heme. The position of the Soret band (407 nm), its high molar extinction coefficient determined with the hemochromogen method (74, 75) (> 130 $mM^{-1}cm^{-1}$), and the charge transfer band at ~632 nm all favor the water-bound complex over the 5c form (105) and suggest that the distal pocket stabilizes the exogenous 6c species. Below pH 5 and at the concentration used for the experiment (~10 μM), the protein precipitates without having completed its shallow transition to the aquomet state. (C) Ferric Y34F S2SbHbN at pH 4.8 (magenta), 7.1 (green), and 10.8 (blue). (D) Absorbance at 404 nm as a function of pH. The colored arrows correspond to the pH of the spectra in (C). Y34F S2SbHbN remains in the aquomet state over a large range of pH.

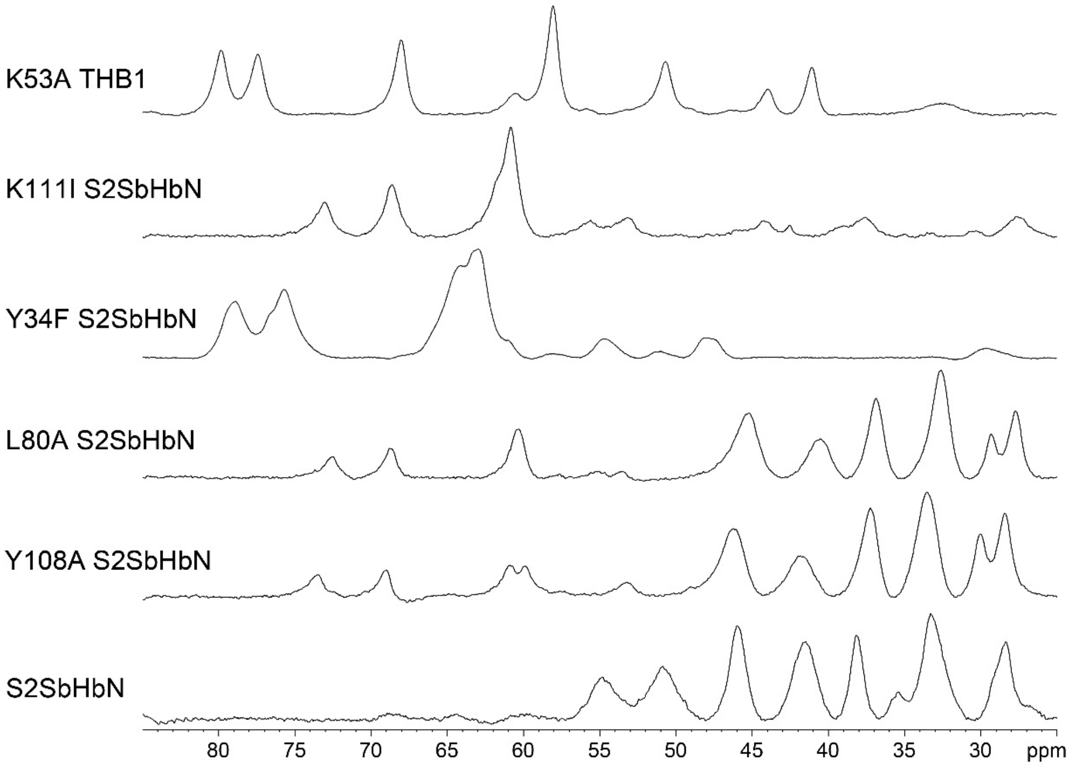

Figure S4 Downfield hyperfine shifted region of the ^{1}H NMR spectra of ferric S2SbHbN and variants (25 °C and pH 7.0–7.5). S2SbHbN has chemical shifts consistent with a spin equilibrium (5/2–1/2) whereas Y34F S2SbHbN has features of a high spin aquomet complex (5/2). The spectrum of *Chlamydomonas reinhardtii* aquomet K53A THB1 is included on top for reference (44). L80A and Y108A S2SbHbN are mostly in the hydroxymet state whereas K111I S2SbHbN is mostly in the aquomet state. Signals were not detected upfield of −20 ppm in any of the spectra. The NMR data are consistent with the interpretation of electronic absorption data (Figure S2). Data collected at 600 MHz; vertical scale arbitrary.

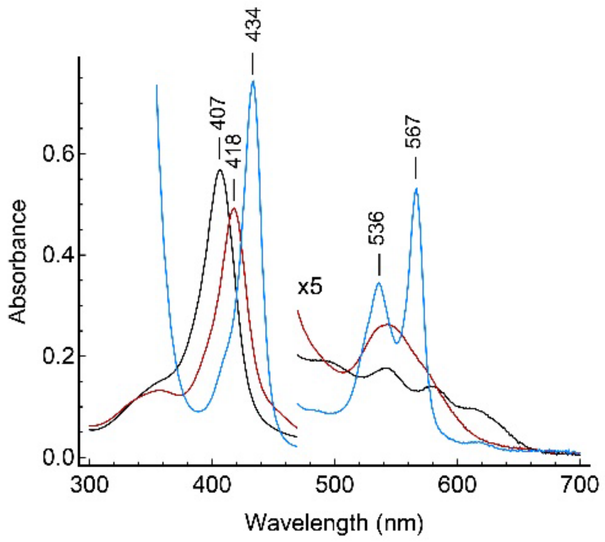

Figure S5 Electronic absorption spectra of S2SbHbN in the presence of cyanide. The ferric protein (black) associates with cyanide (red) and produces a low-spin adduct (blue) with characteristic α and β bands when reduced with excess dithionite. Absorption maxima of the ferrous cyanide adduct (434, 536, 567 nm) are similar to those of the ferrous cyanide adduct of *C. reinhardtii* THB1 (432, 534, 564 nm) (44).

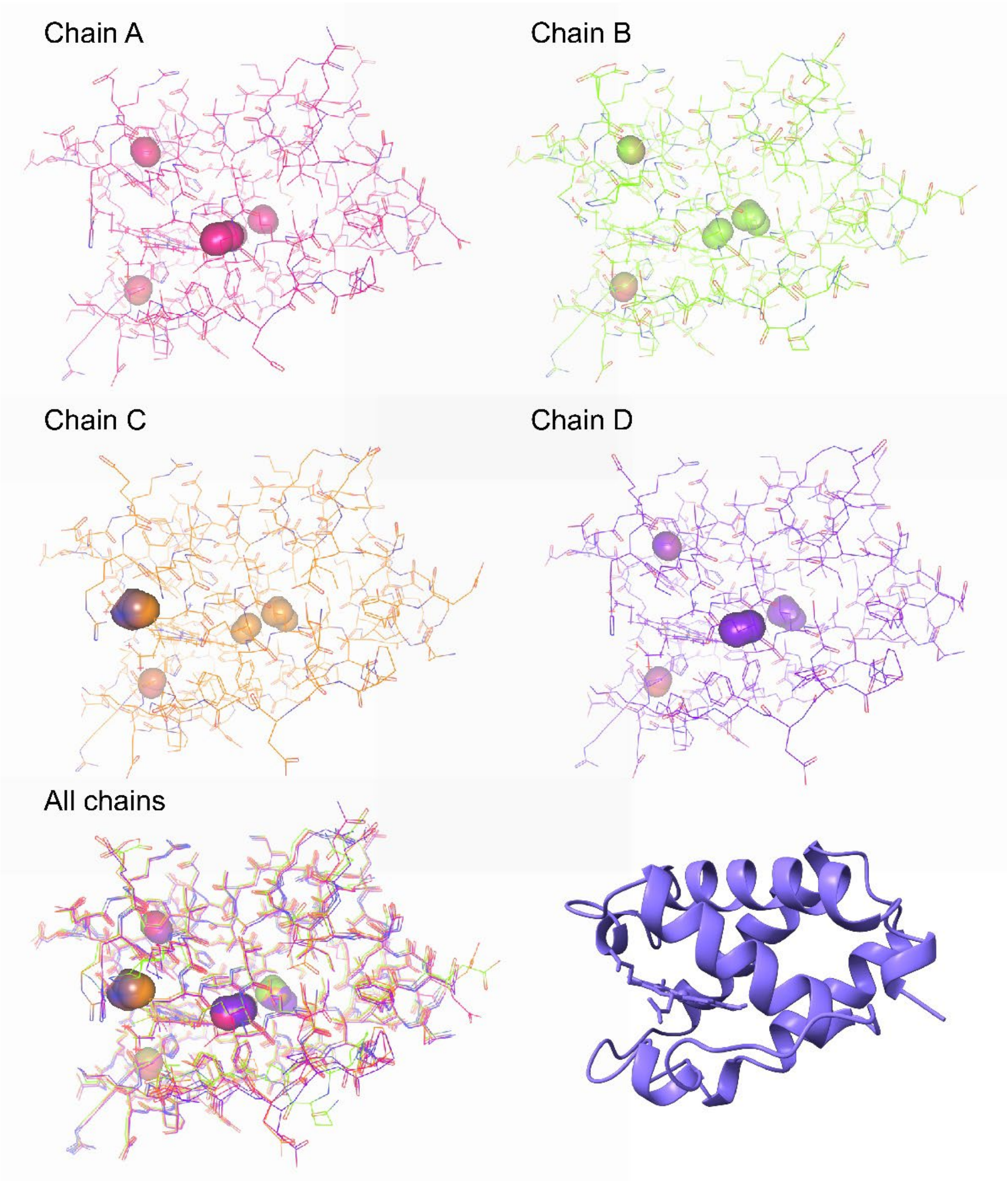

Figure S6 Cavities calculated with Caver Analyst (54) using default radius parameters (minimum radius of 1.4 Å; maximum radius of 3.0 Å) and 5,000 samples. The four chains of structure 8UGZ are shown, along with a superposition ("All chains") and a ribbon diagram for orientation. In all, there are 5 cavities systematically identified in the crystallographic tetramer. The volume of the voids averages 123 ± 15 Å^3 per chain.

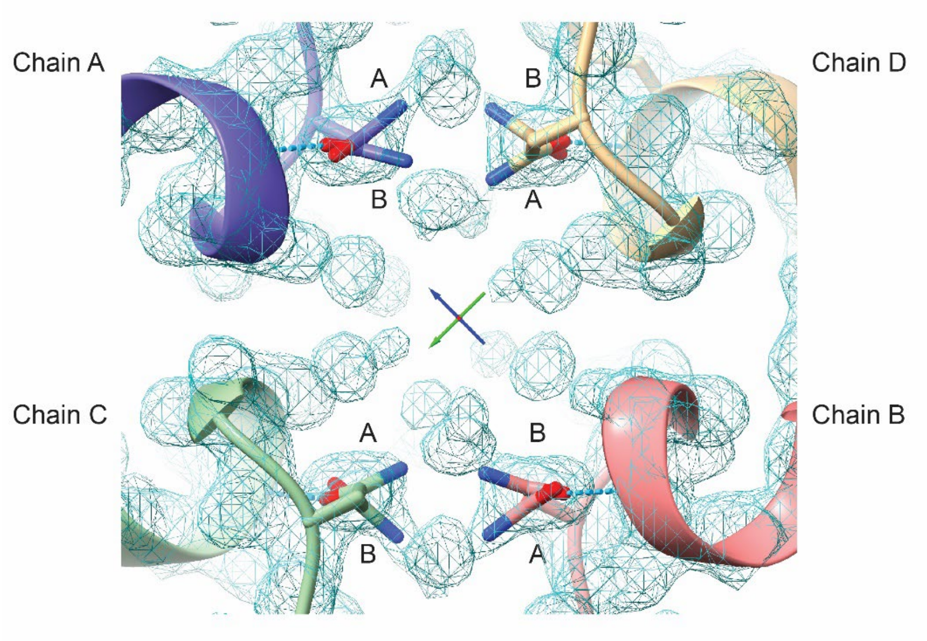

Figure S7 $2mF_o - DF_c$ electron density contoured at 1.0 σ showing where the four chains meet in the asymmetric unit. Only residues Asn75 are represented with sticks. Conformations A and B point so as to prevent clashes. PDB ID 8UGZ, solved at 1.70 Å resolution. The image was produced with ChimeraX (80).

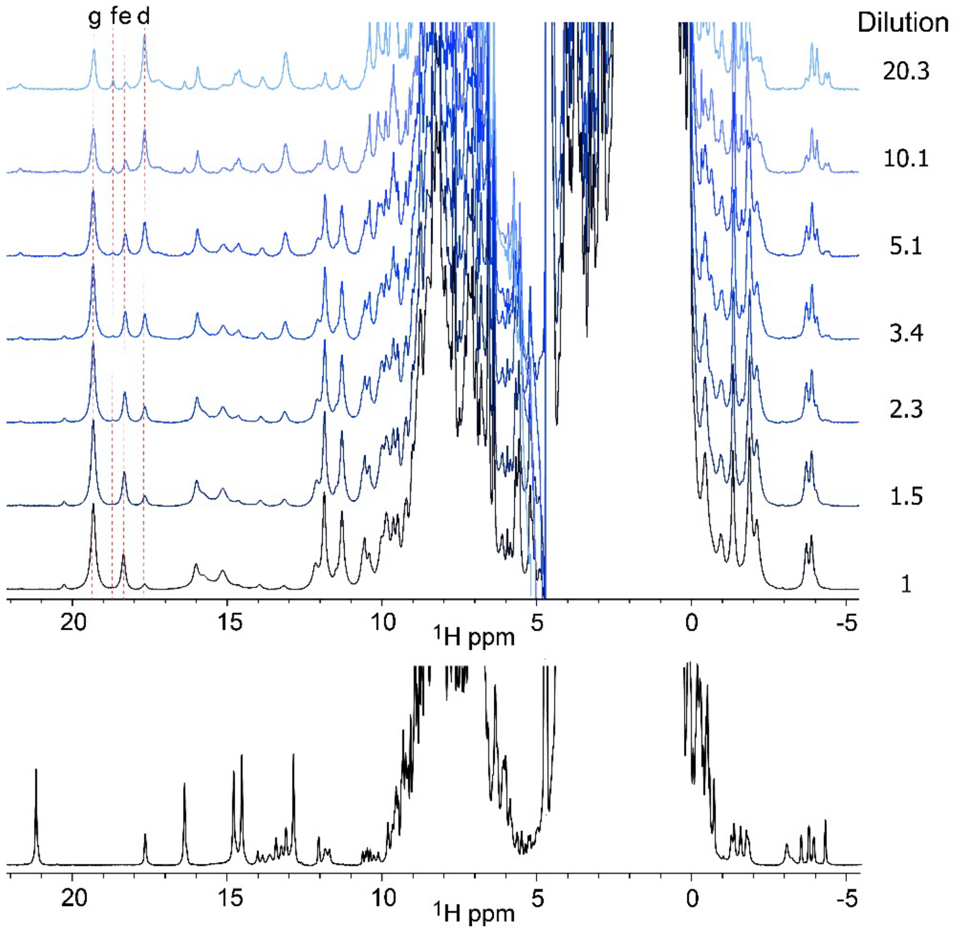

Figure S8 1D ^{1}H NMR spectra of cyanomet S2SbHbN at variable protein concentration. The initial high concentration sample (~ 5 mM, black trace) was diluted stepwise by the indicated factor to low concentration (light blue trace) with additional sample buffer of 20 mM potassium phosphate, pH ~7.5, 90% ^1H_2O/10% ^2H_2O, with KCN maintained at 2-fold excess. Several hyperfine-shifted heme proton resonances were observed to change intensity as a function of protein concentration, indicating self-association behavior (peaks d and e are α-vinyl protons of systems with upfield β pairs a and c in Figure S9; the α proton of system b is unresolved from heme methyl g). The upfield region of the same spectra is shown in Figure 8. All spectra were collected at 600 MHz and 25 °C and processed with 5-Hz line broadening.

For linewidth comparison, the bottom spectrum is that of a highly soluble monomeric protein of the same molecular weight (consensus GlbN in the cyanomet state (106)). Spectrum collected on a ~2 mM sample (600 MHz, 25 °C, processed with 5-Hz line broadening). Vertical scale is arbitrary.

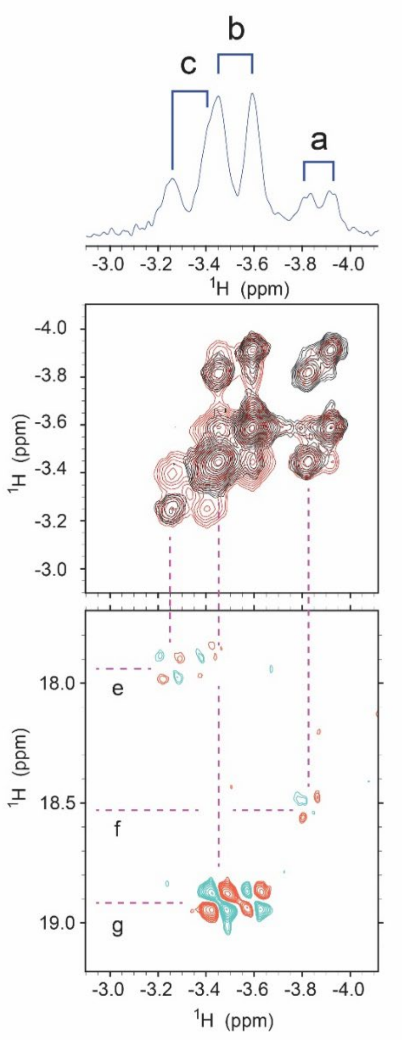

Figure S9 Homonuclear NOESY and TOCSY data for the vinyl β protons shown in Figures 8 and 10 of the text. The NOESY data (red, 75 ms mixing) emphasize intramolecular connectivities whereas the TOCY data (black, 45 ms mixing) emphasize exchange between two states. DQF-COSY data are shown below and place the coupled α protons in the downfield window expected for this low-spin complex. In each set, the DQF-COSY cross peak to the *trans* β proton is stronger than to the *cis* β proton (dashed vertical lines). Sample conditions were ~0.75 mM heme, 3 mM KCN, 20 mM potassium phosphate, pH ~7.5, and 10% ^2H_2O. Data collected at 600 MHz and 35 °C.

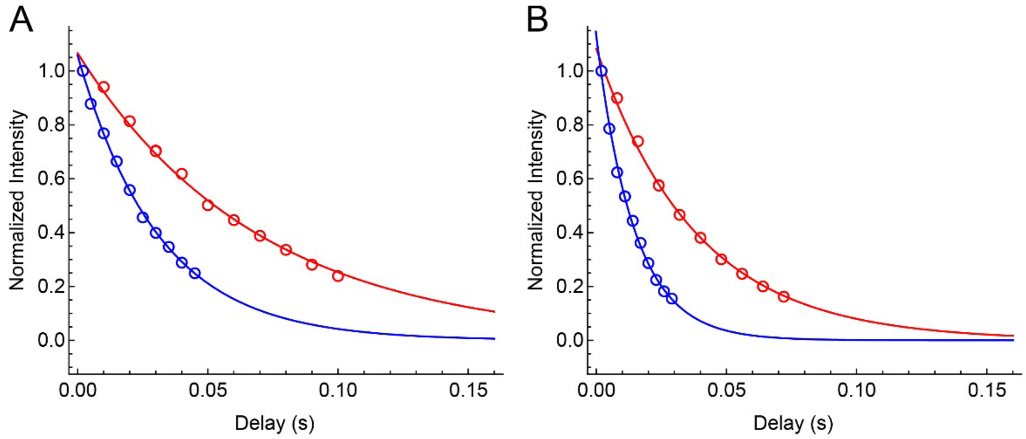

Figure S10 Normalized intensity curves from ^{1}H amide region of 1D [^{15}N,^{1}H]-TRACT spectra (62) of cyanomet S2SbHbN at low and high concentrations. The exponential decay fits shown in the red and blue solid lines correspond to α- and β-spin state relaxation rates, respectively, used to calculate effective rotational correlation times. (A) At 0.2 mM, $R_{\alpha} = 14.4 \pm 0.3$ Hz and $R_{\beta} = 32.2 \pm 0.5$ Hz, yielding a τ_c of ~9.6 ns. (B) At 6.1 mM, $R_{\alpha} = 26.1 \pm 0.5$ Hz and $R_{\beta} = 69.0 \pm 1.3$ Hz, yielding a τ_c of ~24 ns. The pure monomer is expected to have a rotational correlation time of ~7 ns. Sample conditions were 20 mM potassium phosphate, pH 7.7, and 10% $^{2}H_2O$. Data collected at 600 MHz and 25 °C.

The increase in solution viscosity due to the ~30-fold increase in monomer concentration can be estimated with a spherical approximation and the Mooney equation (107, 108): $\eta = \eta_o \exp([\eta]c/(1-(k/s)[\eta]c))$, where η is the solution viscosity, η_o is the pure solvent viscosity (taken to be 0.89 cP at 25 °C), k is the inverse of the packing parameter, set to 1.35, s is the Simha parameter set to 2.5, and c is the concentration in g/L. The low and high viscosities in the TRACT experiments are 0.90 cP and 1.24 cP, respectively, using an intrinsic viscosity [η] of 0.00357 L/g estimated with HydroPro (109) with the structure 8UGZ, chain A. Assuming that proportionality between correlation time and viscosity holds, the monomer would have a τ_c value under 10 ns at the higher concentration. The dimer has an expected correlation time of 16 ns at low concentration and would rise to ~22 ns at the higher concentration.

Additional TRACT data on monomeric and self-associating TrHbs are in reference (110).

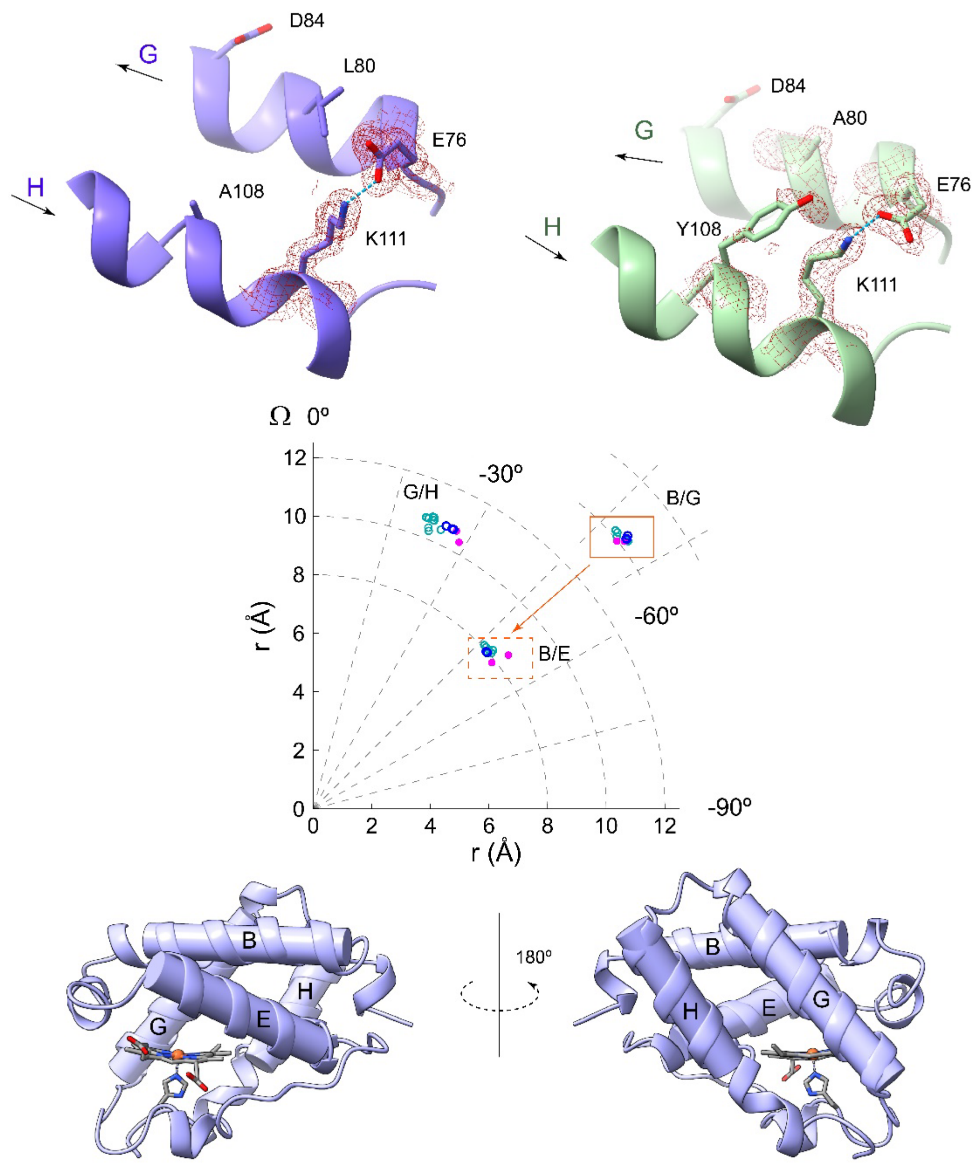

Figure S11 Top: Interaction of Lys111 with Glu76 in structure (left) 8TLS (cyanomet Y108A S2SbHbN, chain A) and (right) 8UZU (cyanomet L80A S2SbHbN, chain C). Electron density (*2mFo−DFc*) is contoured at 1.2 σ. Note that the density does not define Y108 in 8UZU. This figure is for comparison with the G–H interface between the A and C chains of S2SbHbN shown in Figure 9. Middle: Polar plot of helical crossings characteristic of the globin fold (111). Green circles are for structures 8UGZ (cyanomet S2SbHbN), 8VIJ (cyanomet Y34F S2SbHbN), and 8VSH (S2SbHbN with heme d), which have the tetrameric arrangement shown in Figure 7 and self-associate weakly in solution. Filled magenta circles are for 8TLS (cyanomet Y108A S2SbHbN, chains A and B) and blue circles are for 8UZU (cyanomet L80A S2SbHbN, chains A, B, C, D), which are monomeric in solution. The data reflect the reorientation of the G/H and B/E crossings in the interface variants. Values were calculated with ChimeraX. Bottom: the crossings illustrated with chain A of structure 8TLS.

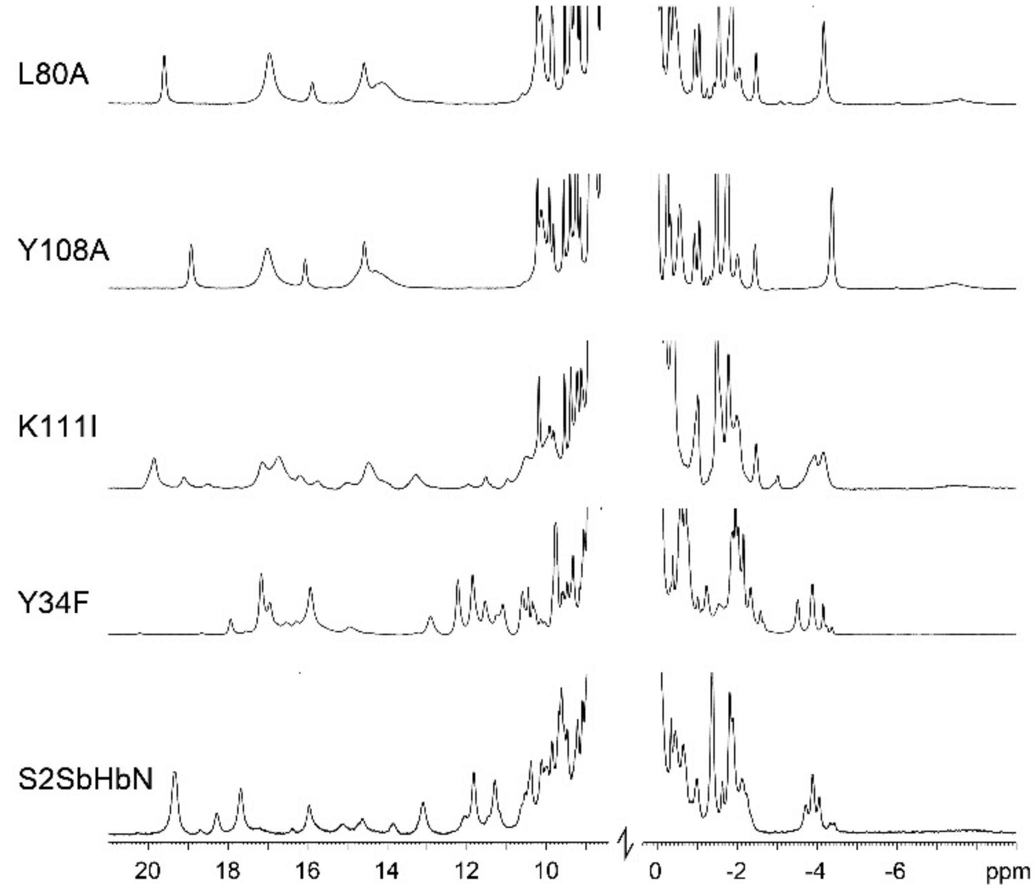

Figure S12 ^{1}H NMR spectra of cyanomet S2SbHbN and variants (600 MHz, 25 °C, pH 7.0–7.5, 10% D_2O, 1 mM heme). The spectrum of S2SbHbN likely reflects the presence of monomer, dimer and higher order species. L80A and Y108A S2SbHbN have simplified spectra compared to S2SbHbN and Y34F S2SbHbN, whereas K111I S2SbHbN does not.

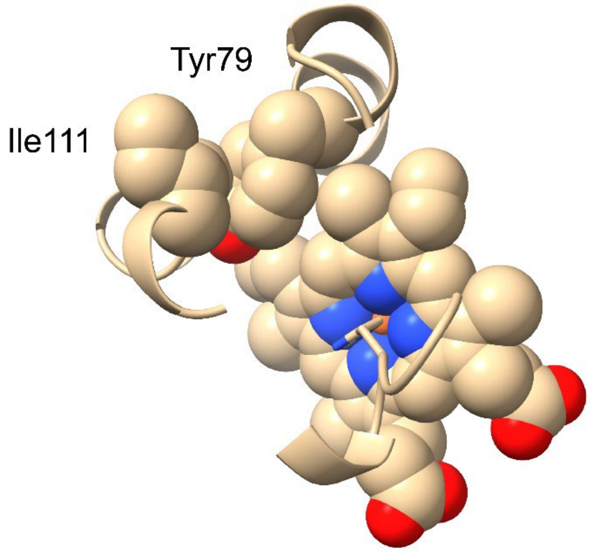

Figure S13 A model of cyanomet K111I S2SbHbN shows the proximity of Ile(H15)111 to Tyr(G5)79 and the heme group. The proximal histidine is included in sticks for orientation. The K111I replacement was made using the structure 8UGZ and ChimeraX. The contacts between Ile(H15)111 and Tyr(G5)79 may explain the effect of the replacement on the heme coordination appearing in Figure S2.

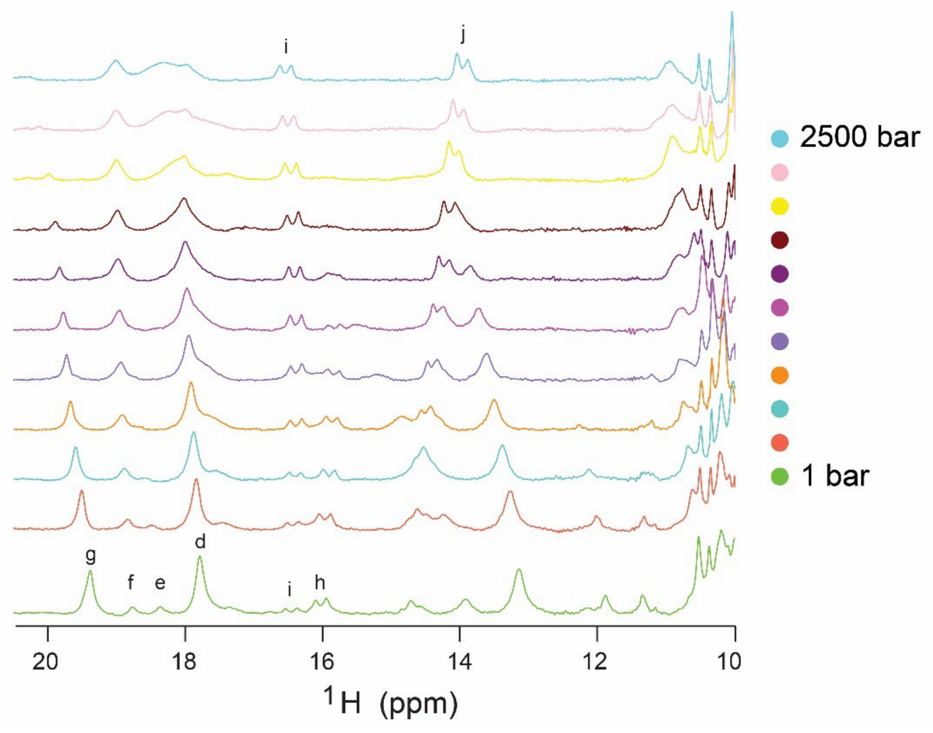

Figure S14 Downfield region of 1D ^{1}H NMR spectra of ^{15}N-labeled cyanomet S2SbHbN at variable hydrostatic pressure (~0.8 mM in 14.12 mM Tris-HCl, 5.88 mM potassium phosphate, pH 7.55, 4 mM KCN, and 90% ^1H_2O/10% ^2H_2O). Each 250-bar step from ambient pressure to 2.5 kbar was allowed to equilibrate for at least 15 min prior to NMR parameter optimization and data collection. The region contains heme resonances, backbone NH resonances near 10 ppm, and resonances attributed to H-bonded HD1 from His69 and His24 (i and j at the highest pressure). The upfield region of the same spectra is shown in Figure 10. Spectra were collected at 600 MHz and 25 °C.

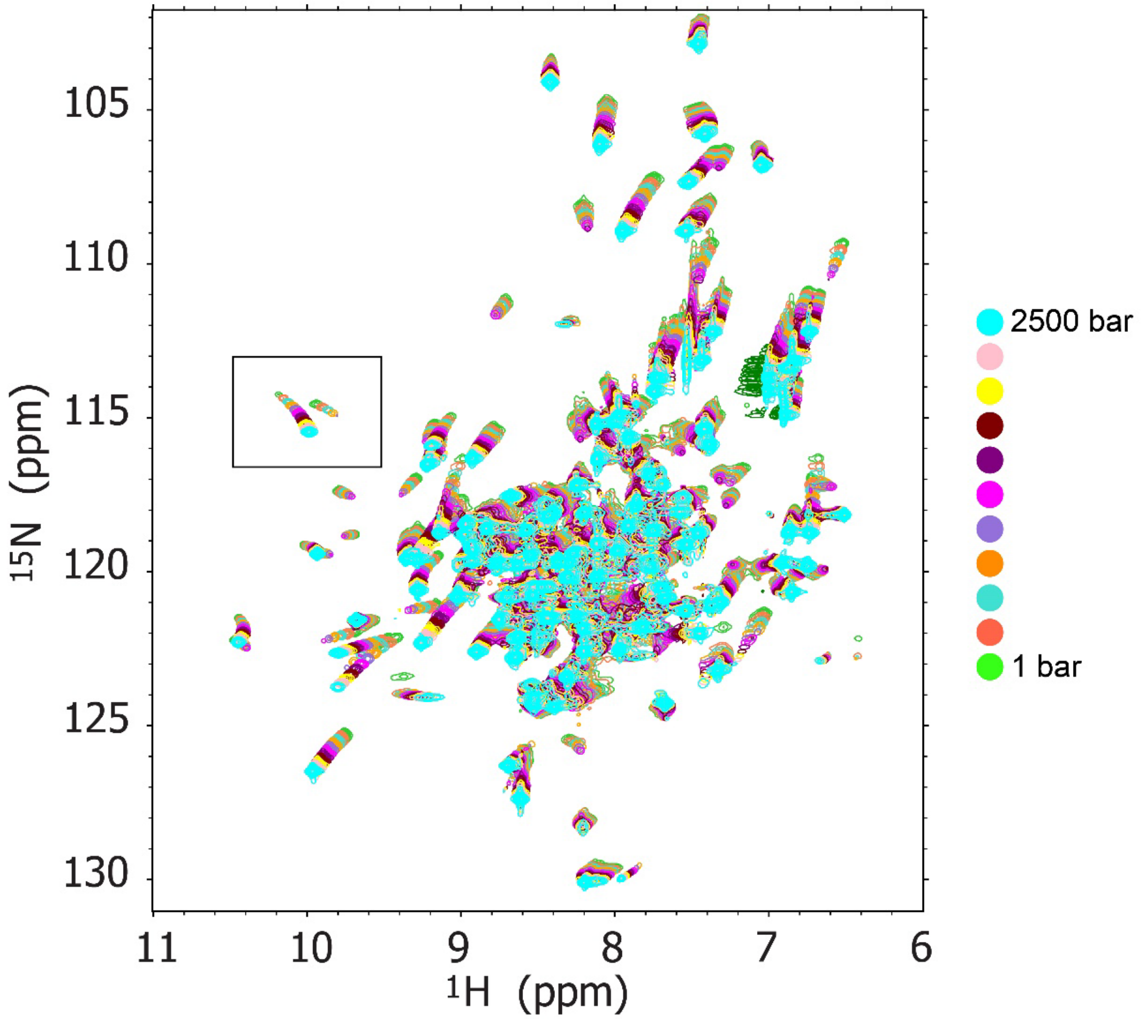

Figure S15 ^{15}N-^{1}H HSQC spectra at variable hydrostatic pressure corresponding to Figure S14. Many cross peaks simply drift, some linearly, some not, but a few, particularly affected by association either disappear or appear as the pressure changes (e.g., signals in the box). The spectrum sharpens and contains fewer peaks at the higher pressure. Spectra were collected at 600 MHz and 25 °C.

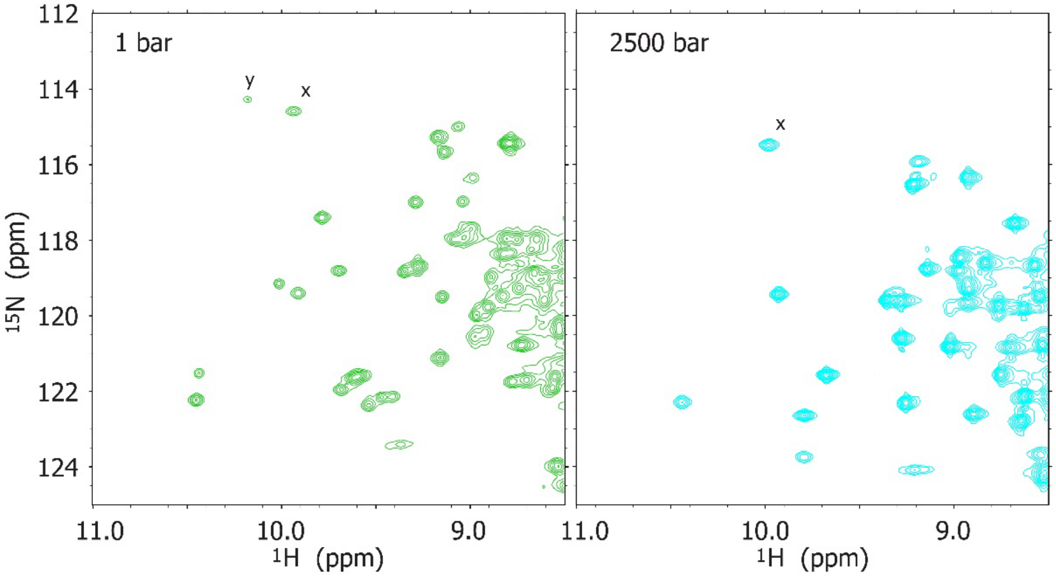

Figure S16 Portion of the ^{15}N-^{1}H HSQC spectra shown in Figure S15 to emphasize the shift in population of folded species (e.g., x and y) with pressure.

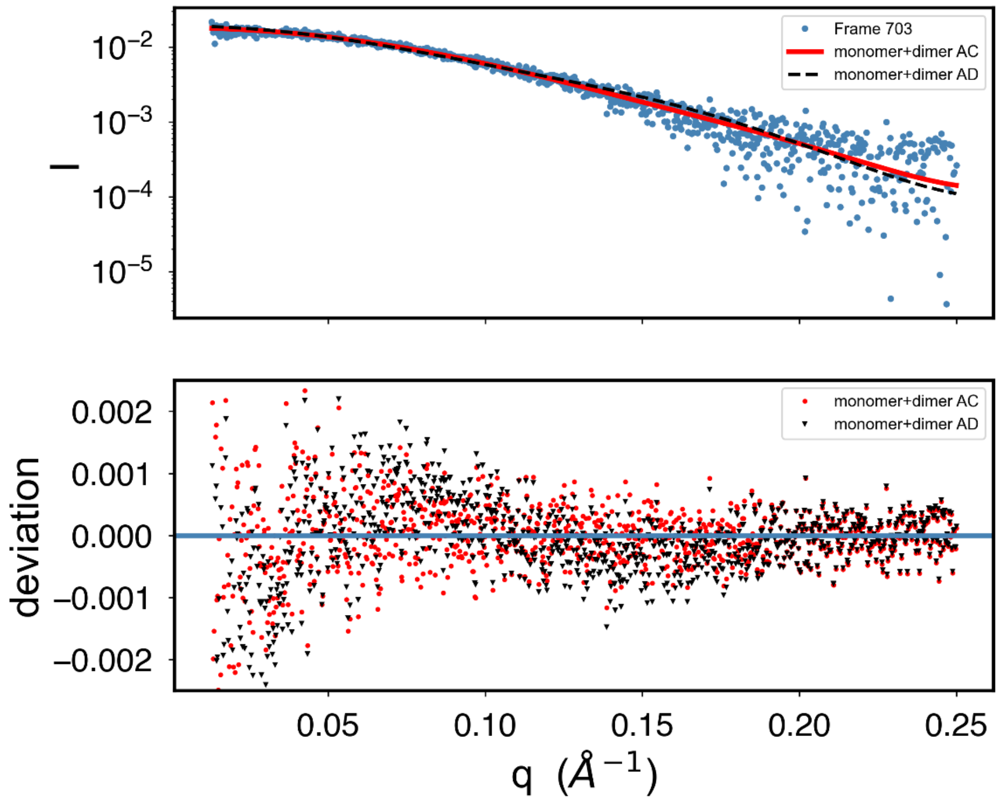

Figure S17 Comparison of scattering profiles for two candidate dimer structures with the experimental scattering intensity (blue dots). The dimer composed of chains A+C from the crystallographic unit cell of PDB 7TT9 produces a 40.7% monomer, 59.3% dimer mixture with goodness-of-fit χ^2 = 1.01 (red). The dimer composed of chains A+D produces a 20.7% monomer, 71.4% dimer mixture with χ^2 = 1.48 (black).

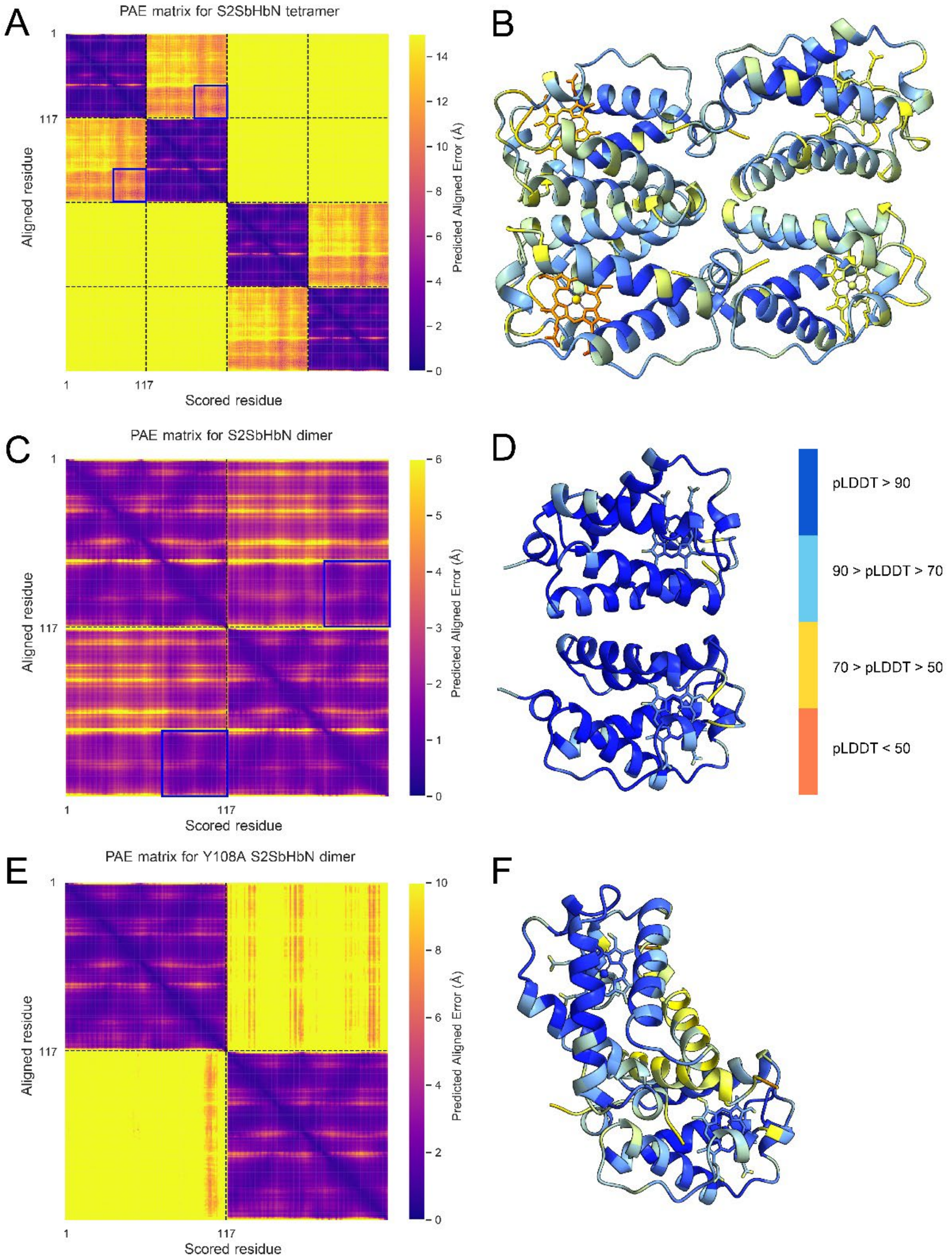

Figure S18 Caption on the next page.

The predictions of AlphaFold-Multimer (68) for (A, B) the S2SbHbN tetramer, (C, D) the S2SbHbN dimer, and (E, F) the Y108A S2SbHbN dimer. The Expected Position Error (or Predicted Aligned Error, PAE) in Å is plotted in (A), (C), and (E) for the best of five results with the indicated color scale to the right of the matrix. The calculations included the heme group, which was removed from the plots for clarity. The blue squares mark the G–H interfaces. The corresponding structural models are shown in (B), (D), and (F) with colors matching the predicted local distance difference test (pLDDT) indicated by the color bar next to (D). The confidence categories are "very high" (pLDDT > 90), "confident" (90 > pLDDT > 70), "low" (70 > pLDDT > 50), and "very low" (pLDDT < 50). The S2SbHbN tetramer prediction (A, B) shows the G–H interface and an A interface (in contrast, the crystallographic interface involves the C–FG side of the G–H dimer). The interface predicted template modelling (ipTM) score and the predicted template modelling (pTM) score are 0.38 and 0.46, respectively, which implies "likely wrong." The S2SbHbN dimer prediction (C, D) anticipates the G–H interface and returns high ipTM and pTM scores of 0.94 and 0.95, respectively, considered "confident high-quality predictions" and supporting the plausibility of the model. For Y108A S2SbHbN, requesting a dimer prediction led to yet a different interface and inconclusive ipTM and pTM scores of 0.61 and 0.69, respectively. The L80A and K111I variants are predicted to form the S2SbHbN G–H dimer with high confidence. The Table below lists values for the five models of each protein discussed in the text.

AlphaFold-Multimer predictions: scores for the five top models

Model	0		1		2		3		4	
S2SbHbN Protein	ipTM	pTM	ipTM	pTM	ipTM	pTM	ipTM	pTM	ipTM	pTM
tetramer	0.38	0.46	0.38	0.45	0.38	0.46	0.37	0.45	0.37	0.45
dimer	0.94	0.95	0.94	0.95	0.94	0.95	0.94	0.95	0.94	0.95
Y108A dimer	0.61	0.69	0.60	0.68	0.59	0.68	0.59	0.67	0.52	0.60
L80A dimer	0.92	0.94	0.92	0.93	0.91	0.93	0.91	0.93	0.91	0.93
K111I dimer	0.94	0.95	0.94	0.95	0.93	0.94	0.93	0.94	0.93	0.94

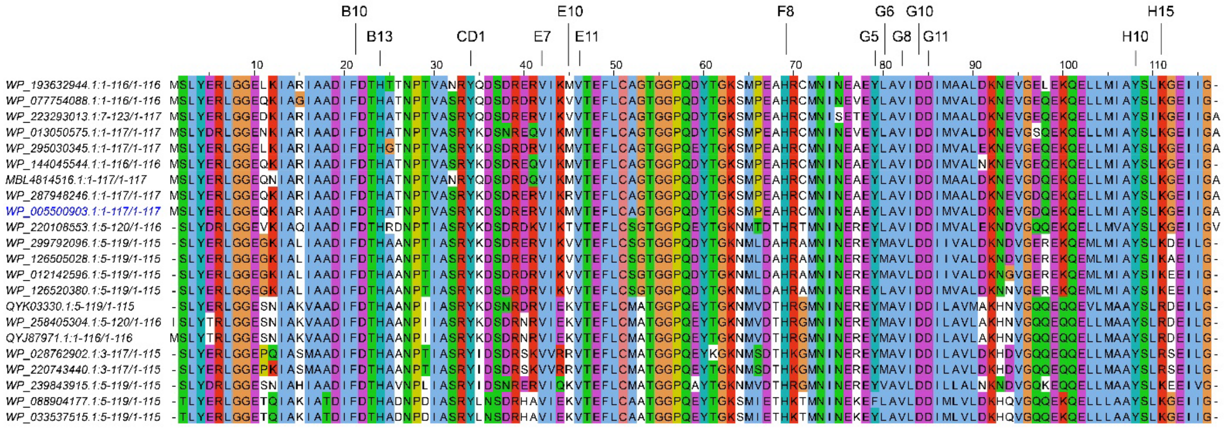

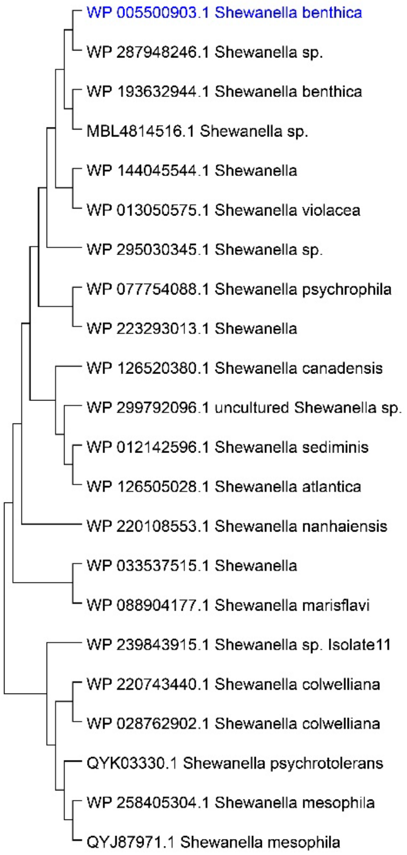

Figure S19 Top: Alignment of the twenty-one *Shewanella* sequences retrieved with a BLAST search using SbHbN as a query (entry in blue). The lowest pairwise identity of the group is 67% over a length of 115/117. WP_193632944.1 *Shewanella benthica* ATCC:43992, DSM:8812; WP_077754088.1 *Shewanella psychrophila* CGMCC:1.6159, JCM:13876;

WP_223293013.1 *Shewanella* Multispecies; WP_013050575.1 *Shewanella violacea* DSS12; WP_295030345.1 *Shewanella* sp.; WP_144045544.1 *Shewanella*; MBL4814516.1 *Shewanella* sp.; WP_287948246.1 *Shewanella* sp.; WP_005500903.1 *Shewanella benthica* KT99; WP_220108553.1 *Shewanella nanhaiensis* KCTC:82799, MCCC:1K06091; WP_299792096.1 uncultured *Shewanella* sp.; WP_126505028.1 *Shewanella atlantica* CCUG:54554, NCIMB:14239; WP_012142596.1 *Shewanella sediminis* HAW-EB3; WP_126520380.1 *Shewanella canadensis* CCUG:54553, NCIMB:14238; QYK03330.1 *Shewanella psychrotolerans* GDMCC:1.2398, KCTC:82649; WP_258405304.1 *Shewanella mesophila* GDMCC:1.2346, KCTC:82640; QYJ87971.1 *Shewanella mesophila* GDMCC:1.2346, KCTC:82640; WP_028762902.1 *Shewanella colwelliana* ATCC 39565; WP_220743440.1 *Shewanella colwelliana* ATCC BAA-642; WP_239843915.1 *Shewanella* sp. Isolate11; WP_088904177.1 *Shewanella marisflavi* JCM:12192, KCCM:41822; WP_033537515.1 *Shewanella* Multispecies. Search performed with NCBI BLAST (112); alignment with Clustal Omega (113); representation with Jalview (95). Bottom: Neighbor joining tree calculated with Mega11 (114).

Among the sources listed, strains of *Shewanella benthica*, *violacea*, *psychrophila*, *colwelliana*, *atlantica*, *nanhaiensis*, *mesophila*, and *marisflavi* also have genes coding for a TrHbO related to that in *S. benthica* (A9D041).

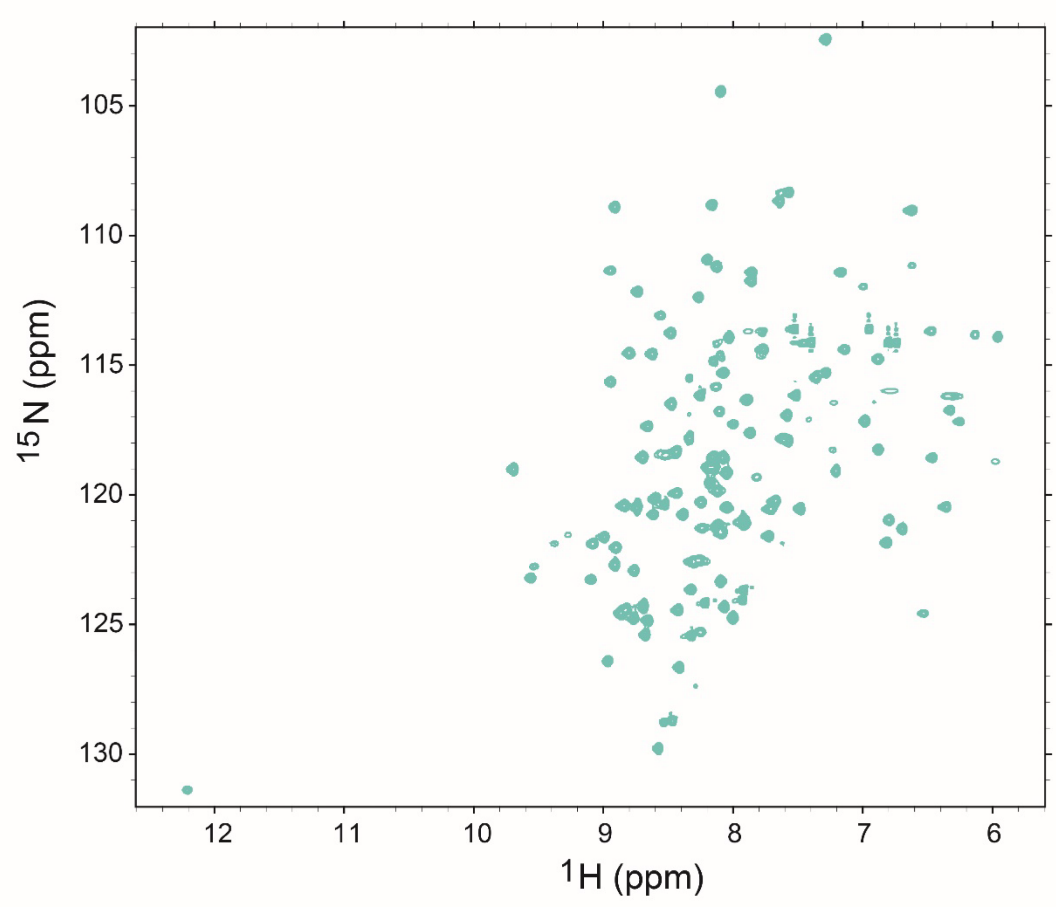

Figure S20 The ^{15}N-^{1}H HSQC spectrum of aquomet THB1 at 2.5 kbar shows that this *Chlamydomonas reinhardtii* protein remains folded at high pressure. Data collected at pH 6.7, 600 MHz and 25 °C (71).

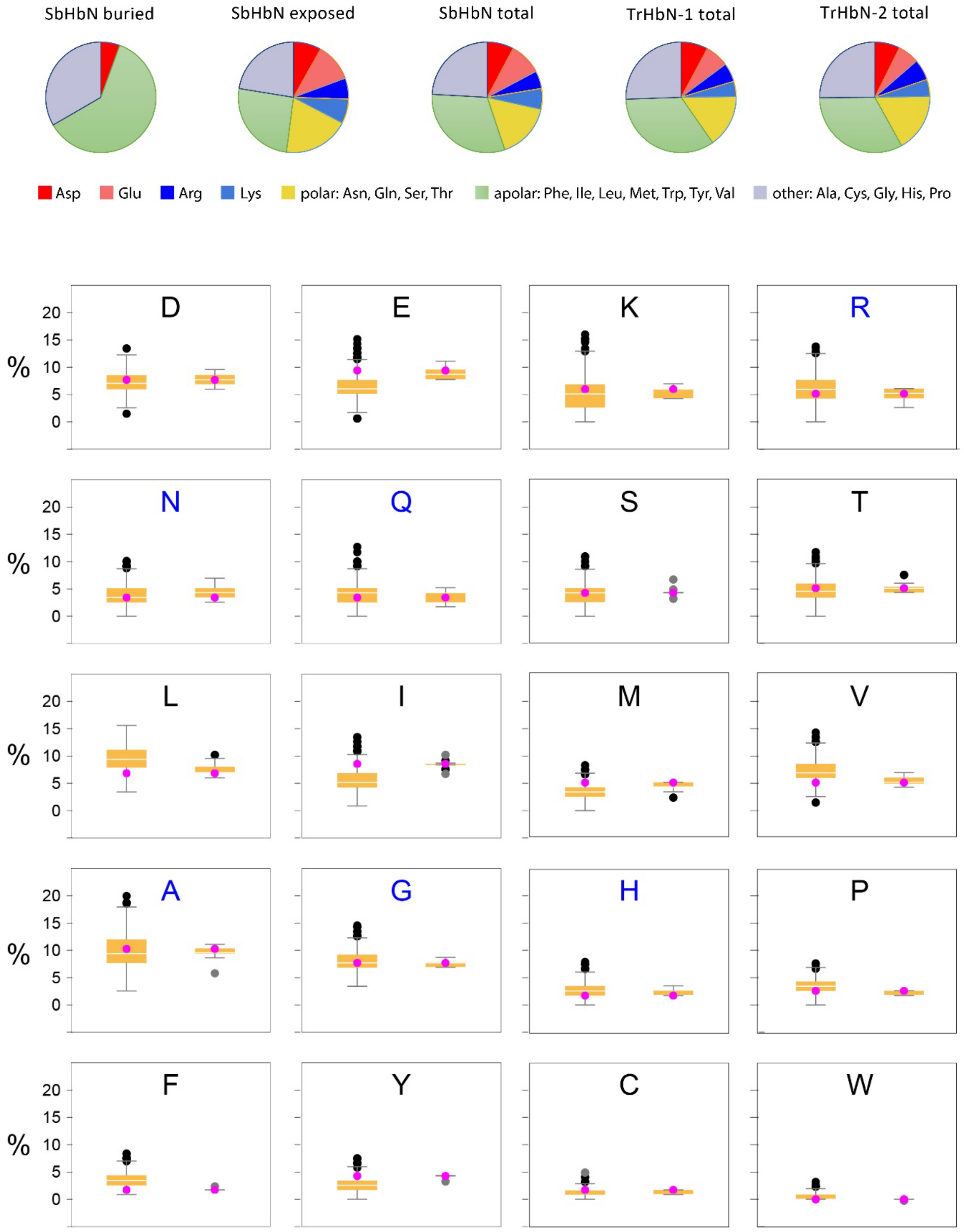

Figure S21 Top: Amino acid composition of TrHbNs. For SbHbN, the structure 8UGZ (chain A) served to determine which residues area on the surface. The Bio.PDB.SASA module of Biopython 1.84 (115) was used to perform the calculation of holoprotein accessible

surface area with the Shrake-Rupley algorithm (116). For each residue type, the reference value of Miller et al. (117) was applied to obtain the fraction of the surface exposed to solvent. A threshold of 5% exposure was chosen to assign residue properties. SbHbN has 18 buried residues and 98 surface residues. The total values are listed in Table S6. The classification of Fukuchi et al. (118) was used. Bottom: Whisker plots of residue percentages in each sequence of (left) the TrHbN-2 MSA (1572sequences) and (right) the *Shewanella* MSA (SbHbN excluded, 21 sequences). The boxes are bound by the 25% and 75% quartiles; the white line is the median. Black dots are outliers (1.5 × interquartile range); gray dots are far outliers (3.0 × interquartile range). Magenta dots indicate the percentages for SbHbN. PP residues (A, G, H, N, Q, R; see Table S6) are in blue font.

References

15. Nath, A., and Subbiah, K. (2016) Insights into the molecular basis of piezophilic adaptation: Extraction of piezophilic signatures. *J. Theor. Biol.* **390**, 117–126
33. Martinez Grundman, J. E., Schultz, T. D., Schlessman, J. L., Liu, K., Johnson, E. A., and Lecomte, J. T. J. (2024) Heme *d* formation in a *Shewanella benthica* hemoglobin. *J. Inorg. Biochem.* **259**, 112654
38. Vuletich, D. A., and Lecomte, J. T. J. (2006) A phylogenetic and structural analysis of truncated hemoglobins. *J. Mol. Evol.* **62**, 196–210
44. Johnson, E. A., Rice, S. L., Preimesberger, M. R., Nye, D. B., Gilevicius, L., Wenke, B. B., Brown, J. M., Witman, G. B., and Lecomte, J. T. J. (2014) Characterization of THB1, a *Chlamydomonas reinhardtii* truncated hemoglobin: linkage to nitrogen metabolism and identification of lysine as the distal heme ligand. *Biochemistry*. **53**, 4573–4589
54. Chovancova, E., Pavelka, A., Benes, P., Strnad, O., Brezovsky, J., Kozlikova, B., Gora, A., Sustr, V., Klvana, M., Medek, P., and others (2012) CAVER 3.0: a tool for the analysis of transport pathways in dynamic protein structures. *PLoS Comput. Biol.* **8**, e1002708
62. Lee, D., Hilty, C., Wider, G., and Wüthrich, K. (2006) Effective rotational correlation times of proteins from NMR relaxation interference. *J. Magn. Reson.* **178**, 72–76
68. Abramson, J., Adler, J., Dunger, J., Evans, R., Green, T., Pritzel, A., Ronneberger, O., Willmore, L., Ballard, A. J., Bambrick, J., Bodenstein, S. W., Evans, D. A., Hung, C.-C., O'Neill, M., Reiman, D., Tunyasuvunakool, K., Wu, Z., Žemgulytė, A., Arvaniti, E., Beattie, C., Bertolli, O., Bridgland, A., Cherepanov, A., Congreve, M., Cowen-Rivers, A. I., Cowie, A., Figurnov, M., Fuchs, F. B., Gladman, H., Jain, R., Khan, Y. A., Low, C. M. R., Perlin, K., Potapenko, A., Savy, P., Singh, S., Stecula, A., Thillaisundaram, A., Tong, C., Yakneen, S., Zhong, E. D., Zielinski, M., Žídek, A., Bapst, V., Kohli, P., Jaderberg, M., Hassabis, D., and Jumper, J. M. (2024) Accurate structure prediction of biomolecular interactions with AlphaFold 3. *Nature*. **630**, 493–500
71. Preimesberger, M. R., Majumdar, A., and Lecomte, J. T. J. (2017) Dynamics of lysine as a heme axial ligand: NMR analysis of the *Chlamydomonas reinhardtii* hemoglobin THB1. *Biochemistry*. **56**, 551–569
74. Berry, E. A., and Trumpower, B. L. (1987) Simultaneous determination of hemes *a*, *b*, and *c* from pyridine hemochrome spectra. *Anal. Biochem.* **161**, 1–15
75. Barr, I., and Guo, F. (2015) Pyridine hemochromagen assay for determining the concentration of heme in purified protein solutions. *Bio-protocol*. **5**, e1594–e1594
80. Pettersen, E. F., Goddard, T. D., Huang, C. C., Meng, E. C., Couch, G. S., Croll, T. I., Morris, J. H., and Ferrin, T. E. (2021) UCSF ChimeraX: Structure visualization for researchers, educators, and developers. *Protein Sci.* **30**, 70–82
95. Waterhouse, A. M., Procter, J. B., Martin, D. M., Clamp, M., and Barton, G. J. (2009) Jalview Version 2--a multiple sequence alignment editor and analysis workbench. *Bioinformatics*. **25**, 1189–91
96. Camacho, C., Coulouris, G., Avagyan, V., Ma, N., Papadopoulos, J., Bealer, K., and Madden, T. L. (2009) BLAST+: architecture and applications. *BMC Bioinformatics*. **10**, 1–9
97. Pei, J., and Grishin, N. V. (2014) PROMALS3D: Multiple protein sequence alignment enhanced with evolutionary and three-dimensional structural information. in *Multiple*

Sequence Alignment Methods, pp. 263–271, Methods in Molecular Biology, Humana Press, Totowa, NJ, **1079**, 263–271

98. Dereeper, A., Guignon, V., Blanc, G., Audic, S., Buffet, S., Chevenet, F., Dufayard, J.-F., Guindon, S., Lefort, V., Lescot, M., and others (2008) Phylogeny. fr: robust phylogenetic analysis for the non-specialist. *Nucleic Acids Res.* **36**, W465–W469
99. Lemoine, F., Correia, D., Lefort, V., Doppelt-Azeroual, O., Mareuil, F., Cohen-Boulakia, S., and Gascuel, O. (2019) NGPhylogeny. fr: new generation phylogenetic services for non-specialists. *Nucleic Acids Res*. **47**, W260–W265
100. Guindon, S., and Gascuel, O. (2003) A simple, fast, and accurate algorithm to estimate large phylogenies by maximum likelihood. *Syst. Biol.* **52**, 696–704
101. Guindon, S., Dufayard, J.-F., Lefort, V., Anisimova, M., Hordijk, W., and Gascuel, O. (2010) New algorithms and methods to estimate maximum-likelihood phylogenies: assessing the performance of PhyML 3.0. *Syst. Biol.* **59**, 307–321
102. Letunic, I., and Bork, P. (2021) Interactive Tree Of Life (iTOL) v5: an online tool for phylogenetic tree display and annotation. *Nucleic Acids Res.* **49**, W293–W296
103. Letunic, I., and Bork, P. (2007) Interactive Tree Of Life (iTOL): an online tool for phylogenetic tree display and annotation. *Bioinformatics*. **23**, 127–128
104. Gasteiger, E., Hoogland, C., Gattiker, A., Duvaud, S., Wilkins, M. R., Appel, R. D., and Bairoch, A. (2005) Protein identification and analysis tools on the ExPASy server. in *The Proteomics Protocols Handbook* (Walker, J. M. ed), Humana Press
105. Shikama, K., and Matsuoka, A. (1989) Spectral properties unique to the myoglobins lacking the usual distal histidine residue. *J. Mol. Biol.* **209**, 489–491
106. Martinez Grundman, J. E., Johnson, E. A., and Lecomte, J. T. J. (2023) Architectural digest: Thermodynamic stability and domain structure of a consensus monomeric globin. *Biophys. J.* **122**, 3117–3132
107. Burckbuchler, V., Mekhloufi, G., Giteau, A. P., Grossiord, J. L., Huille, S., and Agnely, F. (2010) Rheological and syringeability properties of highly concentrated human polyclonal immunoglobulin solutions. *Eur. J. Pharm. Biopharm.* **76**, 351–356
108. Mooney, M. (1951) The viscosity of a concentrated suspension of spherical particles. *J. Coll. Sci.* **6**, 162–170
109. Ortega, A., Amorós, D., and García de la Torre, J. (2011) Prediction of hydrodynamic and other solution properties of rigid proteins from atomic- and residue-level models. *Biophys. J.* **101**, 892–898
110. Nothnagel, H. J., Winer, B. Y., Vuletich, D. A., Pond, M. P., and Lecomte, J. T. J. (2011) Structural properties of 2/2 hemoglobins: The group III protein from *Helicobacter hepaticus*. *IUBMB Life*. **63**, 197–205
111. Lesk, A. M., and Chothia, C. (1980) How different amino acid sequences determine similar protein structures: The structure and evolutionary dynamics of the globins. *J. Mol. Biol.* **136**, 225–270
112. Johnson, M., Zaretskaya, I., Raytselis, Y., Merezhuk, Y., McGinnis, S., and Madden, T. L. (2008) NCBI BLAST: a better web interface. *Nucleic Acids Res.* **36**, W5-9
113. Sievers, F., and Higgins, D. G. (2018) Clustal Omega for making accurate alignments of many protein sequences. *Protein Sci.* **27**, 135–145
114. Tamura, K., Stecher, G., and Kumar, S. (2021) MEGA11: Molecular evolutionary genetics analysis version 11. *Mol. Biol. Evol.* **38**, 3022–3027

115. Cock, P. J. A., Antao, T., Chang, J. T., Chapman, B. A., Cox, C. J., Dalke, A., Friedberg, I., Hamelryck, T., Kauff, F., Wilczynski, B., and de Hoon, M. J. L. (2009) Biopython: freely available Python tools for computational molecular biology and bioinformatics. *Bioinformatics*. **25**, 1422–1423
116. Shrake, A., and Rupley, J. A. (1973) Environment and exposure to solvent of protein atoms. Lysozyme and insulin. *J. Mol. Biol.* **79**, 351–371
117. Miller, S., Janin, J., Lesk, A. M., and Chothia, C. (1987) Interior and surface of monomeric proteins. *J. Mol. Biol.* **196**, 641–656
118. Fukuchi, S., Yoshimune, K., Wakayama, M., Moriguchi, M., and Nishikawa, K. (2003) Unique amino acid composition of proteins in halophilic bacteria. *J. Mol. Biol.* **327**, 347–357
